# A CHO-Based Cell-Free Dual Fluorescence Reporter System for the Straightforward Assessment of Amber Suppression and scFv Functionality

**DOI:** 10.3389/fbioe.2022.873906

**Published:** 2022-04-29

**Authors:** Simon K. Krebs, Nathanaël Rakotoarinoro, Marlitt Stech, Anne Zemella, Stefan Kubick

**Affiliations:** ^1^ Fraunhofer Institute for Cell Therapy and Immunology (IZI), Branch Bioanalytics and Bioprocesses (IZI-BB), Potsdam, Germany; ^2^ Institute for Biotechnology, Technical University of Berlin, Berlin, Germany; ^3^ Institute of Pharmacy, Freie Universität Berlin, Berlin, Germany; ^4^ Institute of Chemistry and Biochemistry, Freie Universität Berlin, Berlin, Germany; ^5^ Faculty of Health Sciences, Joint Faculty of the Brandenburg University of Technology Cottbus - Senftenberg, the Brandenburg Medical School Theodor Fontane and the University of Potsdam, Potsdam, Germany

**Keywords:** expanded genetic code, orthogonal system, noncanonical amino acid, unnatural amino acid, antibody, cell-free protein synthesis, mRFP1, sfGFP

## Abstract

Incorporation of noncanonical amino acids (ncAAs) with bioorthogonal reactive groups by amber suppression allows the generation of synthetic proteins with desired novel properties. Such modified molecules are in high demand for basic research and therapeutic applications such as cancer treatment and *in vivo* imaging. The positioning of the ncAA-responsive codon within the protein’s coding sequence is critical in order to maintain protein function, achieve high yields of ncAA-containing protein, and allow effective conjugation. Cell-free ncAA incorporation is of particular interest due to the open nature of cell-free systems and their concurrent ease of manipulation. In this study, we report a straightforward workflow to inquire ncAA positions in regard to incorporation efficiency and protein functionality in a Chinese hamster ovary (CHO) cell-free system. As a model, the well-established orthogonal translation components *Escherichia coli* tyrosyl-tRNA synthetase (TyrRS) and tRNATyr_CUA_ were used to site-specifically incorporate the ncAA p-azido-l-phenylalanine (AzF) in response to UAG codons. A total of seven ncAA sites within an anti-epidermal growth factor receptor (EGFR) single-chain variable fragment (scFv) N-terminally fused to the red fluorescent protein mRFP1 and C-terminally fused to the green fluorescent protein sfGFP were investigated for ncAA incorporation efficiency and impact on antigen binding. The characterized cell-free dual fluorescence reporter system allows screening for ncAA incorporation sites with high incorporation efficiency that maintain protein activity. It is parallelizable, scalable, and easy to operate. We propose that the established CHO-based cell-free dual fluorescence reporter system can be of particular interest for the development of antibody-drug conjugates (ADCs).

## Introduction

Most proteins found in nature are made up of 20 canonical amino acids (cAA) and are therefore limited in their physiochemical properties and biological functions. The introduction of ncAAs into polypeptides is an emerging technology for the design of synthetic proteins with enhanced characteristics spawning a wide range of novel applications in protein biology, cell biology, and synthetic biology (Li and Liu, 2014). In particular, using amber suppression for the site-specific incorporation of ncAAs with bioorthogonal reactive groups for click chemistry allows precise protein engineering that is superior to traditional labeling techniques such as lysine or cysteine conjugation (Rezhdo et al., 2019; Saleh et al., 2019). The production of ncAA-containing proteins (ncAA-proteins) with an orthogonal tRNA and aminoacyl-tRNA synthetase (aaRS) pair targeting amber stop codons has been accomplished in prokaryotic and eukaryotic cell culture, cell-free systems, and several animals (Brown et al., 2018; Gao et al., 2019).

In recent years, expedient protocols for the identification and generation of compatible tRNA/aaRS/ncAA triplets have been developed (Mukai et al., 2017; Crnković et al., 2019; Cervettini et al., 2020), contributing to an ever growing number of available orthogonal translation systems (OTSs). Previously, investigations on the performance of aaRS and tRNA pairs for ncAA incorporation in a given expression host have mostly been undertaken using fluorescent or enzymatically active reporter proteins located C-terminally of the cognate codon for the orthogonal tRNA. While this allows for sensitive quantification of the total yield of the suppression product, the termination product cannot be quantified, impeding reliable control for sample-to-sample variability and profound OTS characterization (Potts et al., 2020). In an *E. coli* cell-based OTS, Barrick and coworkers addressed this issue by introducing a second fluorescent protein N-terminal to the ncAA-responsive codon. They originated the terms “relative readthrough efficiency” (RRE) and “maximum misincorporation frequency” (MMF), thereby refining the metrics of suppression efficiency (Monk et al., 2017). Based on the same principle, related dual fluorescence reporter assays have been adapted and improved for *Saccharomyces cerevisiae* (Stieglitz et al., 2018), HEK293 cells (Beránek et al., 2018), and CHO cells (Roy et al., 2020). Compared to mass spectrometry (MS), which allows precise quantification and identification of misincorporated amino acids (Mohler et al., 2017), MMF describes an upper bound for cAA misincorporation (Monk et al., 2017).

The most prevailing application of site-specific ncAA incorporation and their conjugation is the development of novel biopharmaceuticals, in particular, for conjugation of a toxin to an antibody (antibody drug conjugates, ADCs) as cancer therapeutic as well as PEGylation of cytokines for improving their half-life in the patient (Kang et al., 2018). Site-specific labeling of proteinaceous therapeutics using ncAAs is superior to nonspecific conjugation techniques as a homogenous product with defined biophysical properties and conjugation site is obtained (Rezhdo et al., 2019). The identification of viable ncAA sites within the sequence of a given protein is critical since the following parameters are highly site-dependent: ncAA incorporation efficiency (Chemla et al., 2018; Cridge et al., 2018; Bartoschek et al., 2021), conjugation efficiency (Reddington et al., 2012; Arpino et al., 2015; Kato et al., 2017), protein solubility, functionality, and stability (Cho et al., 2011; Mu et al., 2012; Shen et al., 2012; Strop et al., 2013; Schinn et al., 2017; Hostetler et al., 2018; Wilding et al., 2018). Despite impressive advances in protein *in silico* structure modeling taking account of ncAA incorporation (Khoury et al., 2014; Singh et al., 2015; Sormanni et al., 2018), these bioinformatic approaches are currently restricted to smaller polypeptides, are often inaccessible to regular wet laboratory personnel, and do not yield reliable functionality prediction of the engineered protein. Therefore, methods for the efficient screening for ncAA-protein activity and for comprehensive OTS characterization are needed (Gao et al., 2019; Rezhdo et al., 2019; Gershenson et al., 2020).

To assist the development of ncAA-containing biopharmaceuticals, we developed a protocol to assess amber positions for ncAA incorporation, conjugation, and activity of ncAA-containing proteins using a CHO cell-free system. Cell-free protein synthesis (CFPS) is a fast and efficient platform technology for the screening and development of ncAA-proteins, allowing straightforward production of tailor-made ncAA-proteins using the protein translation machinery of disintegrated cells (Gao et al., 2019). Most advantageous, CFPS is free of cell-associated constraints such as the cell-wall barrier, often hindering the efficient addition of the ncAA to the translation process. Cell-free systems are most often unaffected by potential cell-toxic effects originating from the introduced orthogonal components or the protein of interest itself. Furthermore, CFPS can be performed using linear templates in high-throughput microliter scale (Dondapati et al., 2020; Silverman et al., 2020). By simply adding an arbitrary orthogonal tRNA/tRNA aminoacyl-synthetase pair, a compatible ncAA with a reactive moiety, and a template with an amber stop codon in its coding sequence to the cell-free reaction, active ncAA-proteins can be produced ready to be conjugated site-specifically to a matching reactive compound without prior purification (Gao et al., 2019; Wu et al., 2020; Stech et al., 2021). Although antibodies are commonly produced in CHO cells (Dalton and Barton, 2014) with *in vivo* production in medium and large scale being more economic compared to CHO-based CFPS (Thaore et al., 2020), we deem practical and economic advantages in using CFPS for initial screening of tRNA/aaRS/ncAA triplets and small scale production for characterization of ncAA-containing proteins.

In this study, we used a CHO cell-free system for the incorporation of the ncAA AzF in a scFv version of the anti-EGFR antibody panitumumab (ABX-EGF, Vectibix®, Amgen, Thousand Oaks, CA, United States). The AzF-tRNATyr_CUA_-TyrRS system has been chosen since it is very well-established, having been used in, for example, CHO cell-based (Sakamoto et al., 2002), *S. cerevisiae* (Nehring et al., 2012), and CHO cell-free OTS (Zemella et al., 2019). The choice for panitumumab scFv as model protein was motivated by the following reasons: 1) The crystal structure of the panitumumab Fab fragment bound to the EGFR extracellular domain III has been published recently (Sickmier et al., 2016), allowing rational elucidation of amber stop codon positions. 2) Most of the ADCs on the market are IgG-based and approved for treating hematological malignancies, while smaller ADC formats are considered beneficial for targeting solid tumors (Deonarain et al., 2018). 3) EGFR is a well-characterized model protein overexpressed in several cancers and readily internalizes upon panitumumab binding (Yewale et al., 2013).

This study provides a proof of concept for time-efficient, high-throughput–compatible ncAA position screening in mRFP1-scFv-sfGFP fusion constructs by using a CHO-based CFPS system. A total of seven panitumumab mRFP1-scFv-sfGFP mutants with individual amber stop codons, two mutants with two amber stop codons, and one mutant with three amber stop codons were assayed for the incorporation of the ncAA AzF, the impact on their antigen binding, and their conjugation to the fluorescent dye DyLight™ 650-Phosphine by Staudinger Ligation. The developed platform allows for the rapid screening of ncAA positioning in regard to amber suppression efficiency and activity. Furthermore, the system can be used for the assessment of the compatibility of novel tRNA/aaRS/ncAA triplets and for the detailed understanding of eukaryotic orthogonal translation system characteristics. In addition, we identified potential ncAA positions for the development of a small format ADC directed against EGFR.

## Results and Discussion

### Framework for CHO-Based Cell-Free Orthogonal Translation System Characterization

**FIGURE 1:**
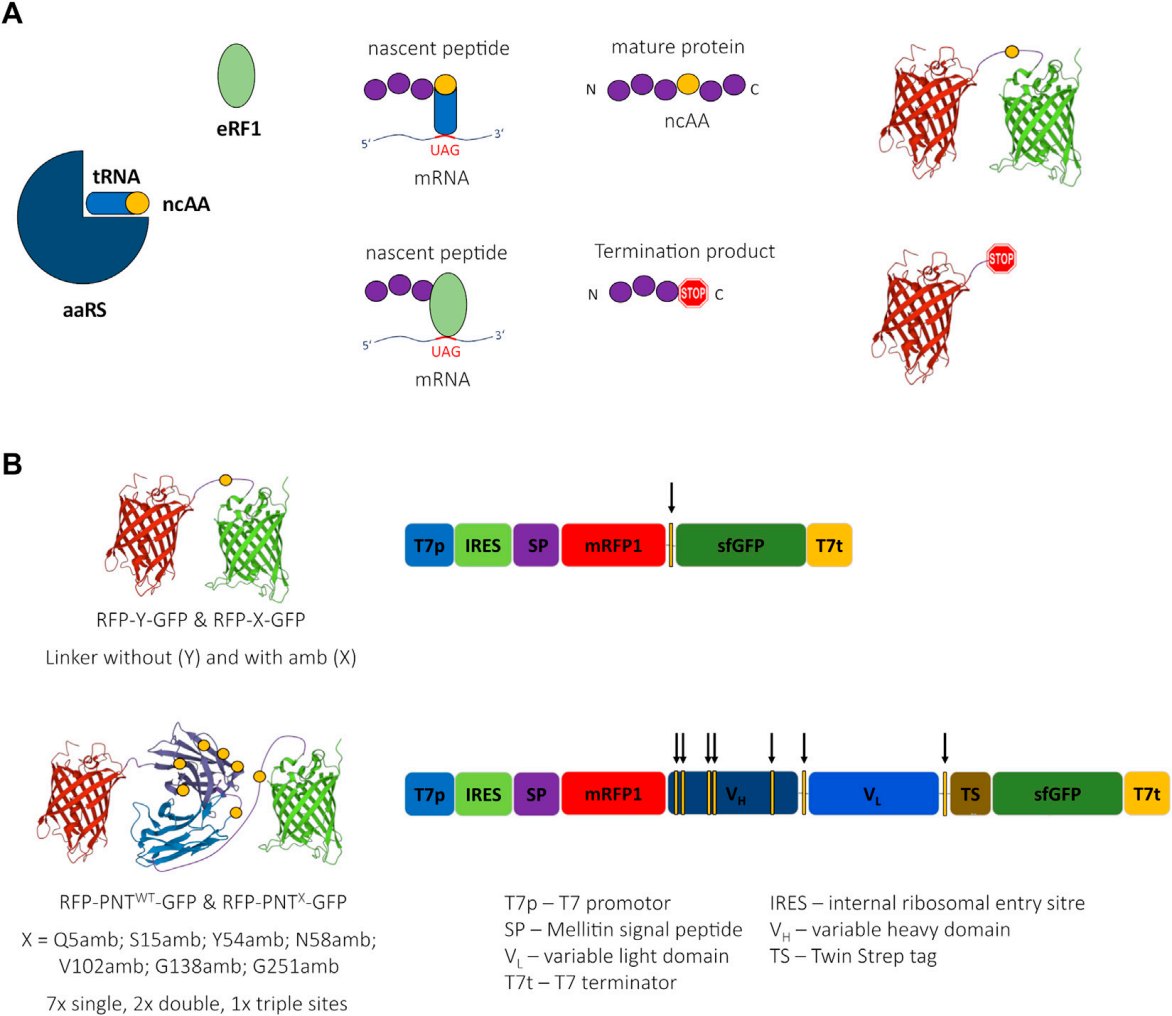
Cartoon of amber suppression, reporter proteins, and ncAA sites. **(A)** Incorporation of a noncanonical amino acid (ncAA) requires three components: The suppressor tRNA is amino-acylated with the ncAA by the aminoacyl tRNA synthetase (aaRS). The ncAA-loaded suppressor tRNA recognizes the UAG (amber) stop codon of the mRNA and the ribosome incorporates the ncAA into the nascent peptide chain leading to a suppression product containing a ncAA. Translation termination competes with ncAA incorporation due to codon stop recognition by release factor eRF1, causing the generation of a termination product not containing a ncAA. **(B)** Schematic 3D-structure of the reporter proteins RFP-Y-GFP and RFP-PNT-GFP (left, the yellow circles depict the ncAA incorporation sites) and schematic of respective DNA sequences (right, the yellow bars with arrows depict the ncAA incorporation sites). 3D structure cartoons are based on PDB structures 2H5Q, 5SX4, and 2B3P.

Orthogonal amber suppression systems allow the incorporation of ncAAs in response to amber stop codons (Figure 1A). We were looking for a high-throughput–compatible, straightforward method that would allow both protein activity assessment depending on ncAA incorporation site and quantification of ncAA incorporation in a scFv. Therefore, we designed a fusion protein of a scFv version of panitumumab (PNT, anti-EGFR) with N-terminal mRFP1 and C-terminal sfGFP (RFP-PNT^WT^-GFP) (Figure 1B). In this fusion protein, amber stop codons were introduced at different positions of the scFv (Table 1). To qualify the system for high-throughput screening applications, cell-free protein synthesis and fluorescence readout were performed in small scale reaction volumes in a 96 well qPCR cycler. In fact, any temperature-controlled plate reader could have been used that is able to accommodate low reaction volumes and detect the appropriate wavelengths for sfGFP and mRFP1 fluorescence.

**TABLE 1:** Nomenclature of panitumumab scFv amber mutants and ncAA location.

Code	Amino acid replaced by AzF	Location
amb5	Q5	V_H_ (FR1)
amb15	S15	V_H_ (FR1)
amb54	Y54	V_H_ (CDR2)
amb58	N58	V_H_ (CDR2)
amb102	V102	V_H_ (CDR3)
amb138	G138	Linker V_H_-V_L_
amb251	G251	Linker V_L_-TS
amb5+15	Q5, S15	V_H_ (FR1)
amb58+102	N58, V102	V_H_ (CDR2), V_H_ (CDR3)
amb5+15+138	Q5, S15, G138	V_H_ (FR1), Linker V_H_-V_L_

Abbreviations: V_H_, heavy chain variable domain; V_L_, light chain variable domain; FR, framework region; CDR, complementary determining region; TS, Twin-Strep tag.

The choice for mRFP1 (Campbell et al., 2002) and sfGFP (Pédelacq et al., 2006) was inspired by the study of Barrick and coworkers, who used N-terminal mRFP1 and C-terminal sfGFP connected with a linker carrying an amber stop codon (RFP-X-GFP) or a Tyrosine (RFP-Y-GFP) at the analogous position (Figure 1B) for screening of different aaRS for ncAA incorporation in proteins expressed in *E. coli* (Monk et al., 2017). The plasmids used here for CFPS include a T7 promotor for mRNA generation and a *Cricket paralysis virus* internal ribosomal entry site (IRES) for cap-independent translation initiation in their 5’ untranslated region (UTR). Furthermore, a melittin signal sequence was added to the N-terminus of the protein and a TwinStrep tag was inserted downstream of the scFv coding sequence (Supplementary Figure S8). The DNA sequence of mRFP1 was changed according to the findings of Whitaker et al. (2015) aiming to silence a potential cryptic ribosome binding site.

### qPCR Cycler-Based Monitoring of Cell-Free Protein Synthesis

**FIGURE 2:**
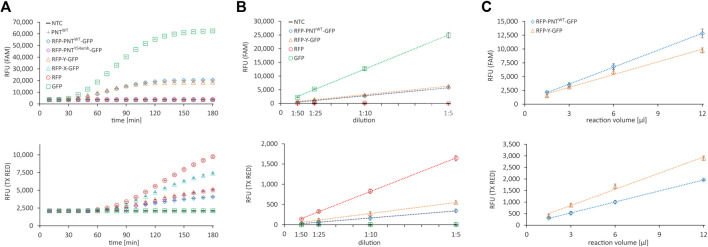
Fluorescence properties of cell-free synthesized reporter proteins. **(A)** Live measurement of fluorescence in the cell-free translation mix of various proteins during cell-free protein synthesis in the FAM channel (top) and the Texas Red channel (bottom). **(B)** Correlation of dilution of the cell-free translation mix and NTC subtracted signal in the FAM channel (top) and the Texas Red channel (bottom). **(C)** Correlation of cell-free translation mix reaction volume and NTC subtracted signal in the FAM channel (top) and the Texas Red channel (bottom). Mean values of triplicates are plotted with error bars representing their standard deviation.

First, we assessed whether the reporter proteins were fluorescently active and whether the signals could be quantified accurately and independently. We performed several cell-free expressions of the mRFP1–sfGFP fusion protein without (RFP-Y-GFP) and with amber stop codon (RFP-X-GFP), the mRFP1-scFv-sfGFP fusion protein without (RFP-PNT^WT^-GFP) and with amber stop codon at position Y54 (RFP-PNT^Y54^-GFP), and the fluorescent proteins or the scFv alone (RFP, GFP, and PNT^WT^). The fluorescence signals of the translation mix of the synthesized proteins in the FAM channel (green, excitation: 450–490 nm, detection: 510–530 nm, FAM) and the Texas Red channel (red, excitation: 560–590 nm, detection: 610–650 nm, TX RED) were recorded in 10 min intervals (Figure 2A). The fluorescence measurements showed that the signal intensity of PNT^WT^ not containing any fluorescent reporter was at a no-template control (NTC) level, while mRFP1 and sfGFP showed an increasing signal over time in the red and green channel, respectively. For all constructs without an amber stop codon in between mRFP1 and sfGFP, a signal was detected in both channels, while constructs with amber stop codon only showed fluorescence in the red channel. This indicated that mRFP1, sfGFP, and fusion proteins thereof were fluorescently active, and cell-free translation was terminated efficiently at the amber stop codon with barely detectable readthrough in the absence of orthogonal components. To confirm these results, we visualized the supernatant (SN1) fraction of the synthesized proteins on a SDS-PAGE autoradiography (Supplementary Figure S1A) and determined protein yields by liquid scintillation counting (Supplementary Figure S1B). The signals in the autoradiograph corresponded to the expected molecular weight, and the protein yields were in a single-digit ng/µl range.

To determine whether the fluorescence signal is proportional to the fluorescent protein amount, the respective cell-free reactions were diluted 1:5, 1:10, 1:25, and 1:50 in PBS and the fluorescence signal was measured in the red and the green channel (Figure 2B). To determine an appropriate working volume for the system, we performed CFPS in volumes from 1.5 to 12 µl with RFP-Y-GFP and RFP-PNT^WT^-GFP and measured the fluorescence after adjusting the volume to 30 µl with PBS (Figure 2C). The fluorescence signals in both channels after subtraction of the NTC value showed linear correlation proportional to dilution and linear correlation nonproportional to reaction volume ≥3 µl. Importantly, within this range, the ratio of green signal to red signal was constant for RFP-Y-GFP and RFP-PNT^WT^-GFP, respectively (Supplementary Figures S2A,B), which represents the numerator for OTS efficiency performance (Monk et al., 2017). The findings indicate that for diluted suspensions, fluorescence detection in our system is accurate at least in boundaries between 50 RFU and 6,000 RFU and reactions can be downscaled to 3 µl. In brief, we found that all proteins could be expressed in the CHO cell-free expression system, fluorescent reporter proteins were active, and their signal could be accurately quantified in a qPCR cycler.

We noted dissimilar green-to-red RFU ratios for reactions with the same template but different incubation times (Supplementary Figures S2A,B). Furthermore, during CFPS, the green fluorescence signal reached a plateau much earlier than the red fluorescence signal (Figure 2A). We reasoned this to be due to slower maturation of the mRFP1 protein. To confirm, we added RNase at different time points during cell-free synthesis (40, 100, and 180 min) and monitored the fluorescence signal over 10 h to be able to differentiate between protein translation and folding (Supplementary Figure S3A, data shown for mRFP1 and sfGFP only). Addition of RNase in the concentration applied leads to degradation of mRNA, thereby aborting protein translation, while fluorescent protein maturation continues (Macdonald et al., 2012). Samples with RNase added after 100 and 180 min display a signal intensity similar to samples without RNase addition. In contrast, samples with RNase added after 40 min show a significantly lower signal. Based on the data, we speculate that for all proteins synthesized here, the resources for translation are exhausted after roughly 2 h and the following increase in fluorescence in the red channel is due to the rather slow maturation of mRFP1. Thus, in our cell-free system run at 30°C, translation and maturation of the sfGFP require approximately 3 h, while mRFP1 maturation is still ongoing but largely completed after 6 h. Based on the results, we decided to extend the reaction time to 6 h to ensure complete mRFP1 maturation, thereby obtaining a maximum red signal and a more stable green-to-red RFU ratio for the proteins carrying N- and C-terminal reporter proteins (Supplementary Figure S3B).

### Functional Analysis of scFv Carrying Dual Fluorescence Reporter Proteins

**FIGURE 3:**
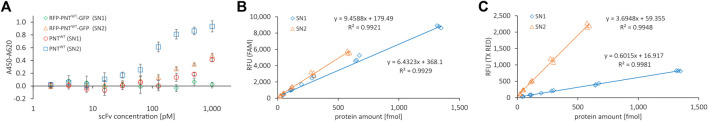
ELISA binding curve of reporter RFP-PNT^WT^-GFP and correlation of fluorescence signal with protein amount determined by liquid scintillation. **(A)** Indirect ELISA binding curve for anti-EGFR scFv with (RFP-PNT^WT^-GFP) and without (PNT^WT^) N- and C-terminal fluorescent reporter proteins against EGFR. PNT^WT^ protein amount per well was normalized according to yields determined by liquid scintillation. Mean values were derived from sample triplicates by subtracting the signal of wells without antigen from the signal of wells with antigen. Error bars represent the standard deviation. **(B)** FAM channel and **(C)** Texas Red channel fluorescence emitted per fmol of protein for RFP-PNT^WT^-GFP from SN1 and SN2 fractions.

CHO lysates used for CFPS within this study allow for the synthesis of highly active antibodies that can be extracted from endoplasmic reticulum (ER)-derived microsomes (Stech et al., 2017). Utilizing a signal peptide from the honey bee toxin melittin, a fraction of the cell-free synthesized proteins is translocated into the ER-derived microsomes and can be released by n-Dodecyl β-D-maltoside (DDM) disruption. To qualify whether the scFv retains its ability for EGFR antigen binding despite carrying N- and C-terminal protein fusions, we synthesized the scFv without (PNT^WT^) and with (RFP-PNT^WT^-GFP) fluorescent reporter proteins (Supplementary Figure S4 for PAGE). We performed an indirect ELISA with serially diluted scFv of the soluble fraction of the cell-free reaction not translocated into the microsomes (supernatant 1, SN1) and of the soluble fraction of the microsome-translocated proteins after microsome disintegration with DDM (supernatant 2, SN2) (Figure 3A). To normalize the molar concentration of cell-free synthesized scFvs in ELISA based on RFU, we established a standard curve for RFP-PNT^WT^-GFP by correlating green and red channel RFU with the protein amount as determined by liquid scintillation counting, respectively (Figures 3B,C; Supplementary Figure S5). Both the red and the green fluorescence signal correlated well with the protein yield, allowing protein yield determination based on RFU signal for the scFv carrying N- and C-terminal fluorescent reporter proteins. In the indirect ELISA, the naked scFv and the scFv carrying N- and C-terminal reporter proteins show a binding curve indicating specific EGFR antigen binding for scFv extracted from SN2, while SN1-derived scFv shows a reduced ELISA binding curve. In agreement with previous observations (Stech et al., 2017), we conclude that the ER-derived microsomes provide an environment beneficial for correct folding of the cell-free synthesized scFv enabling superior antigen binding of SN2 scFv compared to SN1 scFv. We, therefore, decided to focus on the SN2 fraction for our investigations based on the scFv. However, for setting up appropriate aaRS, tRNA, and ncAA concentrations and for other potential screening tasks, direct measurement of the cell-free translation mix (TM) is most convenient.

### Influence of Orthogonal Component Concentration on Amber Suppression

**FIGURE 4:**
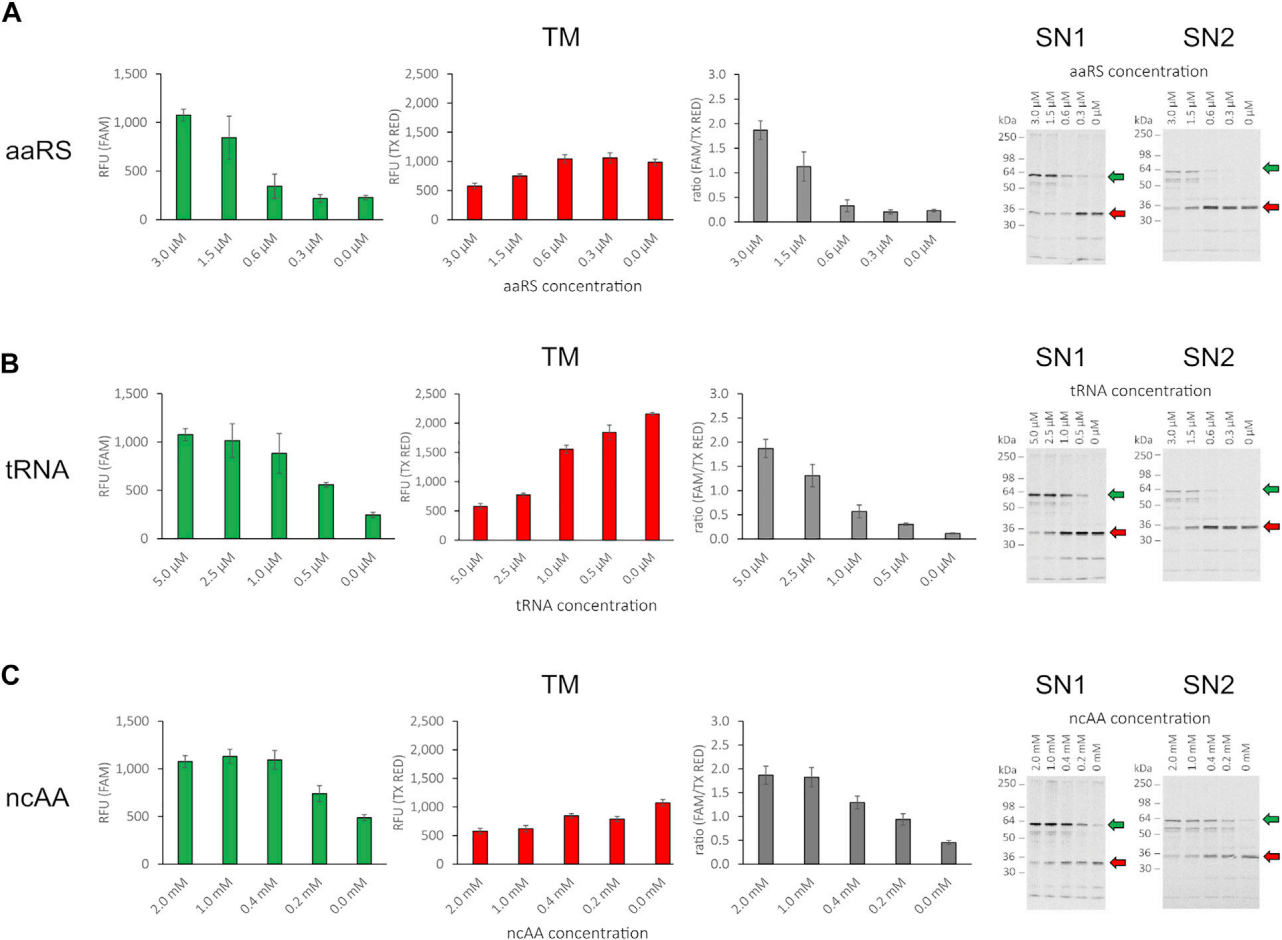
Influence of orthogonal component concentration on fluorescence of RFP-X-GFP reporter protein in the translation mix (TM) and SDS-PAGE of corresponding supernatant fractions SN1 and SN2. **(A)** Variation of aaRS concentration with 5 µM tRNA and 2 mM ncAA, **(B)** variation of tRNA concentration with 3 µM aaRS and 2 mM ncAA, **(C)** variation of ncAA concentration with 3 µM aaRS and 5 µM tRNA for the translation mix (TM) in the FAM channel, the Texas Red channel, ratio of RFU (FAM/TX Red), and the corresponding visualization of the SN1 and SN2 fraction in a radiography of 10% SDS-PAGE (from left to right). Mean values of triplicates were background fluorescence subtracted and are plotted with error bars representing their standard deviation. Green and red arrows depict the full length protein and the termination product, respectively.

The incorporation of the ncAA AzF by the orthogonal aaRS, TyrRS, and tRNATyr_CUA_ pair has been demonstrated previously in CHO-based cell-free systems (Zemella et al., 2019). To assess whether the cell-free amber suppression OTS is functional in our chosen setup and to evaluate the impact of orthogonal component concentration on amber suppression, we synthesized the reporter construct without antibody (RFP-X-GFP) at varying concentrations of ncAA, tRNA, and aaRS. Initially, as limited screening, we chose an arbitrary concentration range for ncAA, tRNA, and aaRS and kept two component concentrations constant (3 µM aaRS, 5 µM tRNA, and 2 mM ncAA, respectively), while varying the third component to investigate the impact on amber suppression in the translation mix. The RFU in the FAM channel and the TX RED channel of the translation mix (TM) were determined and the corresponding supernatant fractions SN1 and SN2 were visualized by SDS-PAGE autoradiography (Figure 4). For all three orthogonal components, a clear dependency on orthogonal component concentration for red and green signal could be observed in the TM. The obtained results correlate well with the signal intensity in the SN1 and SN2 autoradiograph. If one of the orthogonal components was omitted, a strong signal corresponding to the molecular weight of the RFP-X-GFP reporter termination product (30.1 kDa) was observed in the autoradiographs. This was accompanied by a higher red signal and a lower green signal, reflected in a low green-to-red RFU ratio. In contrast, when increasing the concentration of the respective orthogonal component, an increasing strength of the autoradiograph signal corresponding to the molecular weight of the full-length product (58.5 kDa) was observed, in line with an increasing green-to-red ratio. The ratio of the green and red signal projects the amount of suppression product in relation to the termination product as a measure for OTS efficiency: The higher the ratio, the greater the suppression efficiency. The system, therefore, can be used to elucidate the individual impact of the aaRS, tRNA, and ncAA and optimize their concentrations in the reaction mix. Furthermore, fluorescence measurement of the TM allows for assessing the suppression efficiency of SN1 and SN2 fractions.

We conclude that the cell-free OTS is functional and the supplementation of the cell-free reaction with the orthogonal components ncAA, tRNA, and aaRS leads to the incorporation of the ncAA AzF in the nascent polypeptide chain at sites with in-frame amber stop codon allowing translation elongation and synthesis of the downstream sfGFP. Since release factor eRF1 is present in the CHO host strain used for cell-free lysate production, the ncAA-loaded tRNA competes with eRF1 for binding to the cognate UAG anticodon. Therefore, with increasing concentrations of orthogonal components, the competition with eRF1 binding to the amber stop codon is shifted toward orthogonal tRNA binding and concurring ncAA incorporation. In contrast, if one of the orthogonal components is reduced, the equilibrium is shifted toward eRF1 binding, and translation is therefore incrementally terminated at the amber stop codon located between mRFP1 and sfGFP. By individually evaluating the orthogonal components, we observed that the yield of the suppression product is not proportional to the concentration of the orthogonal components added to the cell-free reaction, but reaches a plateau at high concentrations (Figures 4B,C). Furthermore, when comparing readthrough events with one of the orthogonal components missing, as expected for the case where aaRS and tRNA were present, we observed the highest green signal (Figure 4C, 0 mM ncAA). Therefore, since no ncAA is present, misincorporation with a cAA at the amber stop codon took place either by orthogonal or endogenous tRNA recognition. When omitting aaRS or tRNA, the readthrough was significantly reduced. In the case without tRNA, this indicates amber stop codon recognition of an endogenous tRNA (Figure 4B, 0 µM tRNA). In the case without aaRS, the observed readthrough is either due to amber stop codon recognition of the orthogonal tRNA loaded by an endogenous aaRS with any cAA or the ncAA. Alternatively, endogenous tRNA amber stop recognition may also occur in that case (Figure 4A, 0 µM aaRS). The results show that controlling the orthogonal component concentration individually allows for assessment of their respective impact on amber suppression in cell-free systems. However, dual fluorescence reporter systems do not allow for identification of the incorporated residue. This would require more elaborate instrumentation such as MS. MS is a powerful method for precise amber codon responsive amino acid identification and quantification (Mohler et al., 2017) and also allows for high-throughput applications (Yamazoe et al., 2020). Still, apart from the instrumentation, it requires highly trained specialists and is generally laborious and cost-intensive (Zhang and Rockwood, 2015).

The individual influence of orthogonal component concentration on amber suppression or amino acid identity was not subject to further investigation; instead, we focused on determining a working OTS setup. Therefore, different combinations of aaRS and tRNA concentrations with 1 mM of ncAA were analyzed for fluorescence signal in all fractions (TM, SN1, and SN2), and the corresponding SN1 and SN2 fraction were visualized in a PAGE autoradiograph (Supplementary Figure S6). The results affirm the previous observation that TM, SN1, and SN2 fluorescence are interdependent and correlate well to the signals in the SDS-PAGE radiograph. For all conditions tested, we obtained satisfactory yields of the suppression product and decided to proceed using 1.5 µM aaRS, 2.5 µM tRNA, and 1 mM ncAA for further experiments involving the scFv from the SN2 fraction.

### Evaluation of RFP-PNT-GFP Variants Containing Noncanonical Amino Acids

So far, it was shown that the cell-free OTS used here is functional and that amber suppression can be accurately quantified using N- and C-terminal fluorescent reporter proteins. The wildtype scFv specifically binds its antigen with and without N- and C-terminal fluorescent proteins when produced in the ER-derived microsomes. To identify positions in the scFv that allow high suppression efficiency and antigen binding, amber stop codons at seven different sites were introduced by Gibson Assembly to generate 10 different RFP-PNT-GFP variants, including two scFv variants with two ncAA incorporation site and one scFv variant with three ncAA incorporation sites. The positions were chosen according to two criteria: the potential involvement of the native amino acid side chain in EGFR binding, and its accessibility for conjugation based on 3D structure analysis (Table 1 & PDB entry 5SX4).

**FIGURE 5:**
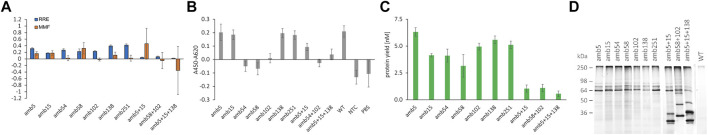
Quantification of RFP-PNT-GFP amber suppression with different ncAA incorporation sites, functional analysis by indirect ELISA, and conjugation to DyLight™ 650-Phosphine. **(A)** RRE and MMF for different ncAA positions in RFP-PNT-GFP extracted from SN2 fraction. **(B)** Functionality of 100 pM of the RFP-PNT-GFP variants containing ncAAs extracted from SN2 fraction by indirect ELISA (OD450-OD620 signal with coated EGFR subtracted by OD450-OD620 signal without coated EGFR). **(C)** Protein yield of full length product in the SN2 fraction, calculated based on FAM signal by using the formula in Figure 3B. **(D)** In-gel fluorescence of SDS-PAGE after conjugation to DyLight™ 650-Phosphine for RFP-PNT-GFP extracted from SN2 fraction containing ncAAs at different sites. The green arrow depicts the full-length product. Mean values are derived from triplicates and their standard deviation is plotted as error bars.

The amber mutants and the wildtype RFP-PNT-GFP scFvs were synthesized either in the presence (+ortho) of 1.5 µM aaRS, 2.5 µM tRNA, and 1 mM ncAA or in their absence (-ortho). ScFvs were extracted from the ER-derived microsomes (SN2 fraction), and the fluorescence signal was detected in a 96 well plate qPCR cycler in the red and green channel (Supplementary Figure S7). From the signal of the red and green channel, the RRE_+ortho_ and RRE_-ortho_ was determined and the MMF was calculated (Figure 5A) for each RFP-PNT-GFP amber mutant using the following formulas:

$$\mathrm{RR}E_{+\mathrm{ortho}}=\frac{{\left(\frac{\text{RFU}\;\text{FA}{\text{M}}_{\mathrm{amb}}}{\text{RFU}\;\text{T}\text{X}\;\text{RE}{\text{D}}_{\mathrm{amb}}}\right)}_{+\mathrm{ortho}}}{{\left(\frac{\text{RFU}\;\text{FA}{\text{M}}_{\mathrm{WT}}}{\text{RFU}\;\text{TX}\;\text{RE}{\text{D}}_{\mathrm{WT}}}\right)}_{+\mathrm{ortho}}};\;\mathrm{RR}E_{-\mathrm{ortho}}=\frac{{\left(\frac{\text{RFU}\;\text{FA}{\text{M}}_{\mathrm{amb}}}{\text{RFU}\;\text{TX}\;\text{RE}{\text{D}}_{\mathrm{amb}}}\right)}_{-\mathrm{ortho}}}{{\left(\frac{\text{RFU}\;\text{FA}{\text{M}}_{\mathrm{WT}}}{\text{RFU}\;\text{TX}\;\text{RE}{\text{D}}_{\mathrm{WT}}}\right)}_{-\mathrm{ortho}}};\;\mathrm{MMF}=\frac{\mathrm{RR}E_{-\mathrm{ortho}}}{\mathrm{RR}E_{+\mathrm{ortho}}}.$$

RRE_+ortho_ values close to 1 indicate an efficient amber suppression, while RRE_+ortho_ values close to 0 indicate a high fraction of termination events at the amber stop codon. High MMF values (up to 1) indicate high misincorporation, while low MMF values (down to 0) indicate low misincorporation. Note that the term MMF used here differs from the definition as originated by Barrick and coworkers (Monk et al., 2017). Monk et al. (2017) calculated MMF based on the RRE without ncAA, thereby describing ncAA incorporation events in the presence of the aaRS and tRNA, while here, MMF is based on the RRE without any orthogonal components, thereby calculating MMF with respect to basal translational readthrough.

The RRE values of the scFvs carrying N- and C-terminal reporter proteins vary according to position (Figure 5A). As expected, constructs carrying more than one amber stop codon show a low RRE close to 0. The RRE of the single mutants lies between 0.17 and 0.42, indicating pronounced differences in incorporation efficiency. Codon context effects and cellular context are hypothesized to be the main determinant for amber suppression efficiency in mammalian cells, but remain widely elusive (Phillips-Jones et al., 1995; Chemla et al., 2018; Cridge et al., 2018). To our knowledge, no data in the literature are available for suppression efficiency neither in CHO cell-free systems nor in CHO cells, which make our data hard to cross-reference. A very recent study developed a linear regression model called iPASS to predict ncAA incorporation efficiencies based on codon context in HEK and mESC cells using the orthogonal aaRS/tRNA pair pyrrolysyl-tRNA synthetase and tRNA^Pyl^_CUA_ pair from *Methanoscarcina* species (Bartoschek et al., 2021). We calculated the iPASS score for the different ncAA incorporation sites and determined a weak positive correlation with RRE except for position G138 (data not shown). Although the iPASS model is an exciting approach for prediction of amber suppression, we expect the iPASS model being valid only for the specific tRNA/aaRS in the specific hosts examined and is likely not transferable to a CHO cell-free system with the given aaRS/tRNA/ncAA triplet.

In the anti-EGFR scFv examined here, the highest suppression efficiency was determined for amber stop codons in place of G138 and G251, which share identical 4 bases downstream of the amber stop codon and are both located in flexible Gly-Ser linkers (Supplementary Figure S8). Furthermore, amber positions N58 and V102 show identical three bases downstream with similar RRE. In contrast, positions Q5 and S15 that also bear identical three downstream bases show differing RRE. Although no general conclusion regarding context effects can be drawn, particular ncAA sites with high suppression efficiency were identified. The MMF determined here shows the lowest values for amber positions 54, 102, and 251, and no correlation with suppression efficiency is observed. Strikingly, even some ncAA codons with identical +4 to +6 bases show considerable differences in readthrough, most notably amb58 vs. amb102 and amb138 vs. amb251. Since no data on either suppression efficiency or basal readthrough are available for CHO cells, the results cannot be cross-referenced to the literature. In fact, for the first time, we report here data on suppression efficiency and readthrough in CHO context.

For assessing antigen binding and conjugation of ncAA-containing scFvs carrying N- and C-terminal fluorescent proteins, we calculated the suppression protein yield (Figure 5C) based on the FAM standard curve for SN2 (Figure 3B) and normalized the antibody molar amount for ELISA to 100 pM. Antibodies containing ncAAs were incubated on an EGFR-coated plate, and StrepTactin-HRP was used to detect the suppression products carrying a TwinStrep tag at the C-terminal part (Figure 5B). The scFv containing two or three ncAA incorporation sites was not normalized due to low yield of the suppression product but was used in 1:6 dilution. According to the expectation, the variants containing a ncAA at distal regions of the antigen are able to bind to EGFR and show a comparable signal to the wildtype antibody: Q5amb, S15amb [on Framework (FR) 1 of V_H_], G138amb (on flexible V_H_-V_L_ linker), and G251amb (on flexible TwinStrep linker). The variants with ncAA positions with direct antigen contact are not able to bind to EGFR, such as Y54amb, N58amb (on V_H_-CDR2), and V102amb (on V_H_-CDR3) (see PDB entry 5SX4). We, therefore, conclude that functionality of the cell-free synthesized scFvs carrying dual fluorescent reporter proteins can be assessed using the presented workflow. While functionality is contingent on amber suppression, despite efficient amber suppression, the resulting suppression product can be inactive, making screening of amber positions for protein activity necessary.

To assess the accessibility of the ncAA in the scFv for click chemistry reaction, DyLight™ 650-Phosphine was conjugated to equal molar amounts of scFv variants by Staudinger ligation and visualized by in-gel fluorescence after SDS-PAGE (Figure 5D). Fluorescence signals were detected in the scFvs which carry an amber stop codon, while for the wildtype scFv, which does not carry an amber stop codon, no fluorescence signal is observed. The expected molecular weight of the full-length scFv fused to mRFP1 and sfGFP is 87.9 kDa, but three bands were detected between 60 and 90 kDa when the samples were not heated before loading onto the gel (Figure 5D). The three bands indicated differently migrating protein species of the full-length RFP-scFv-GFP fusion after LDS addition. In fact, a complete denaturation of the fusion protein required heating of the sample to 95°C, which resulted in a single band at around 90 kDa. Conversely, 10 min incubation at 70°C was not sufficient for complete denaturation of the fusion protein (data not shown). Despite the unexpected running behavior, the results of the in-gel fluorescence gel indicated that the RFP-scFv-GFP fusion construct can be selectively conjugated to a compound with a matching reactive moiety for the ncAA sites investigated and represented a qualitative proof for ncAA incorporation.

## Conclusion

NcAA incorporation into proteins using CFPS gains increasing interest due to the system’s tolerability of cell-toxic substances, its open nature, and flexibility (Gao et al., 2019). Previously, we described the production of antibodies based on the use of CHO lysate containing microsomes derived from the endoplasmic reticulum (Stech et al., 2017). By targeting antibodies to these microsomes with a melittin signal peptide, post-translational modifications such as disulfide bonds and core glycosylation can be achieved. Recently, we reported the incorporation of a ncAA into the CH1 domain of an anti-Muc1 IgG1 and its conjugation to a fluorophore (Stech et al., 2021). Taking this concept further, this study for the first time reports a straightforward CFPS-based dual fluorescence reporter system that allows both quantification of amber suppression and activity of a ncAA-containing scFv. By performing cell-free reactions and measurement in a qPCR cycler, the platform allows parallelization, is scalable to low µl volume, and is easy to operate.

While MS allows for precise amino acid identification at the amber stop codon (Mohler et al., 2017) and continues to evolve for higher throughput with lower sample quantity (Zhang and Rockwood, 2015), the dual fluorescence reporter systems allow straightforward, nondestructive, *in situ* quantification of ncAA incorporation in high-throughput combined with low instrumentation requirements. Dual reporter assays to quantify suppression efficiency and fidelity have been established recently, and their superiority over single reporter systems and ease of use compared to MS are increasingly appreciated (Monk et al., 2017; Stieglitz et al., 2018; Potts et al., 2020; Roy et al., 2020; Bartoschek et al., 2021). While there have been efforts in the past to evaluate the effects of ncAA positioning in a protein of interest, these studies investigated ncAA positions in the reporter itself (Albayrak and Swartz, 2013; Wandrey et al., 2016), were not able to evaluate the protein of interest’s activity (Amiram et al., 2015; Arpino et al., 2015; Hostetler et al., 2018; Stieglitz et al., 2018; Cao et al., 2019; Jakob et al., 2019) or the associated suppression efficiency/fidelity (Cho et al., 2011; Zimmerman et al., 2014; Wu et al., 2015; Schinn et al., 2017). This study demonstrates for the first time a system that allows assessment of suppression efficiency and activity of an antibody containing ncAAs in a single workflow. We used a modular Gibson Assembly strategy, which allows fast and economic template generation with an arbitrary protein of interest and any desired ncAA incorporation sites. Therefore, this platform is of high value for ADC development, a novel class of anti-cancer drugs (Peters and Brown, 2015), whose activity and stability has been shown to strongly depend on the ncAA site (Shen et al., 2012). The system may further be used to screen for compatibility of aaRS, tRNA, and ncAA triplets and facilitate elucidation of codon context effects on suppression efficiency in cell-free systems.

## Materials and Methods

### Gibson Template Generation of Wildtype PNT-scFv Plasmid

The recombinant human single chain antibody fragment PNT-scFv was designed based on sequence information from PDB entry 5SX4 (Sickmier et al., 2016). V_H_ and V_L_ chains were identified using ANARCI (Dunbar and Deane, 2016). V_H_ and V_L_ genes were codon optimized using GeneOptimizer algorithm (Raab et al., 2010) for *Cricetulus griseus* codon usage. Between codon optimized V_H_ and V_L_ DNA sequences, a 60 bp sequence coding for a (G_4_S)_4_ linker was inserted and the construct was synthesized in plasmid pUC57-1.8k by BioCat (Heidelberg, Germany), including 20 bp flanking regions on both the 5′ and 3′ end of the V_H_ and V_L_, respectively (pUC57-1.8k_PNT-scFv). Using Gibson assembly (see later), the V_H_-linker-V_L_ sequence was assembled into plasmid pUC57-1.8 k with a 5′ untranslated region (UTR) containing a T7 promotor sequence to allow for efficient T7 RNA polymerase mediated transcription and an internal ribosomal entry site (IRES from the intergenic region of the Cricket paralysis virus) to allow for initiation factor–independent translation initiation. The coding sequence was N-terminally fused to a melittin signal sequence to translocate *de novo* synthesized polypeptide chains into the lumen of the microsomal vesicles (Brödel et al., 2013). To the 3’ part of the coding sequence, a Twin-Strep-tag separated from the V_L_ sequence by a (G_2_S)_2_-SA linker was added. The resulting plasmid was purified from the Gibson reaction mix using DNA Clean and Concentrator-5 (Zymo Research, Irvine, CA, United States) with elution in 6 µl of ultrapure water. Then, 2 µl solution containing purified plasmid was used to transform electrocompetent *E. coli* JM109, and the plasmid was prepped from an overnight liquid culture of a single clone using QIAprep Spin Miniprep Kit (Qiagen, Venlo, Netherlands). Integrity was checked by restriction digest using *Nhe*I and *Bgl*II (NEB, Ipswich, MA, United States) and Sanger sequencing (LGC Genomics, Berlin, Germany).

### Gibson Primer Design for Introduction of Amber Stop Codons

Generally, the primers listed in Supplementary Table S1 were designed with an annealing temperature of 60 ± 2°C calculated using Oligoanalyzer algorithm (IDT, Leuven, Belgium) with salt and oligo concentration settings according to PCR reaction conditions. The homologous stretch between two linear PCR products required for Gibson Assembly was designed to possess a melting temperature of >60°C. Primers introducing a TAG codon were designed with 15 bp homology upstream and downstream of the TAG codon.

### PCR Reactions, DNA Fragment Gel Extraction, and Gibson Assembly for Introduction of Amber Stop Codons

PCRs were carried out in 25 µl volume using 0.04 ng/μl of plasmid template with Q5® Hot Start High-Fidelity DNA Polymerase (NEB, Ipswich, MA, United States), according to the manufacturer’s recommendations, supplemented with dNTP Mix, PCR Grade (Qiagen, Venlo, Netherlands), and primers listed in Supplementary Table S1 in different combinations (Supplementary Table S2) using a cycling program with an annealing temperature of 60°C and 35 cycles. After addition of 3 µl of 10x FastDigest Green Buffer (Thermo Fisher Scientific, Waltham, MA, United States), 25 µl PCR reaction was separated electrophoretically in 1.2% TBE-agarose gels and target bands were excised and column-purified using Zymoclean Gel DNA Recovery kit (Zymo Research, Irvine, CA, United States) and 10 µl of ultrapure water for elution. DNA concentration was measured using Nanodop-2000c spectrophotometer (Thermo Fisher Scientific, Waltham, MA, United States) to determine the required volume in Gibson assembly reactions. Then, 0.1, 0.3, and 1 pmol of DNA was used in Gibson assembly for fragments with sizes >1,500 bp, between 300 and 1,500 bp, and smaller than 300 bp, respectively. On ice, an equal volume of 2x NEBuilder® HiFi DNA Assembly Master Mix (NEB, Ipswich, MA, United States) was added to the DNA fragments, mixed by pipetting, and incubated for 30 min at 50°C in a Biometra TRIO thermocycler (Analytik Jena, Jena, Germany). The reactions were purified using DNA Clean and Concentrator-5 (Zymo Research, Irvine, CA, United States) with elution in 6 µl of ultrapure water, and 2 µl was used to transform *E. coli* JM109 for plasmid miniprep and sequencing as described previously.

### Gibson Assembly of mRFP1-PNT-sfGFP Amber Mutants

PCR reactions, Gibson assembly, DNA purification, transformation, plasmid miniprep, and sequencing were performed as described previously. The respective plasmids of the amber mutants were amplified using primer Bird1-X-NcoI-PNT_F and Strep2-X-Bird2_R and joined *via* Gibson assembly with the DNA fragment obtained from amplification of pUC5718_NCM+D_mRFP1-Y-sfGFP with primer Bird2_F and Bird1_R to add 5′ mRFP1 and 3’ sfGFP to the coding sequence of the amb mutants.

### Preparation of Orthogonal Components

Orthogonal components were prepared as described previously (Zemella et al., 2019). In brief, pQE2-eAzFRS-SII coding for an *E. coli*-derived tyrosyl-tRNA-synthetase [TyrRS, including the mutations Thr37, Ser182, Ala183, and Arg265 (Chin et al., 2003) with a T5 promotor and a C-terminal StrepII-Tag] was synthesized in RTS 500 ProteoMaster *E. coli* HY Kit (Biotechrabbit, Neuruppin, Germany) on a Thermomix Comfort (Eppendorf, Hamburg, Germany) with RTS 500 thermomixer adapter at 30°C, 1,000 rpm for 24 h using Isopropyl β-d-1-thiogalactopyranoside (f.c. 1 mM). Synthetase was purified using Gravity flow Strep-Tactin® superflow mini-column with Strep-Tactin® Purification Buffer Set (IBA, Göttingen, Germany). Elution buffer was exchanged to synthetase storage buffer (f.c. 50 mM HEPES pH 7.6, 10 mM KOAc, 1 mM MgCl2, 4 mM Dithiothreitol) using Zeba™ Spin Desalting Columns (7K MWCO, 0.5 ml, Thermo Fisher Scientific, Waltham, MA, United States); synthetase was concentrated using Amicon® Ultra Centrifugal Filters (10 K device, 0.5 ml, Sigma-Aldrich, St. Louis, MI, United States), quantified with NanoDrop 2000c spectrophotometer (Thermo Fisher Scientific, Waltham, MA, United States), and stored at −80°C. The template for *in vitro* transcription of tRNATyr_CUA_ (SupF Gene) was prepared by amplifying plasmid pQE2-tRNATyr_CUA_ with tRNA specific primer (Supplementary Table S1) in a PCR reaction [1 × Taq Buffer, 0.2 mM dNTP mix, 2.5 mM MgCl2, 0.04 U/µL Taq DNA polymerase (Qiagen, Venlo, Netherlands), 0.5 µM forward primer, 0.5 µM reverse primer, 0.01 ng/μL plasmid], purified using QIAquick PCR Purification Kit (Qiagen, Venlo, Netherlands), and DNA concentration was quantified using a Nanodop-2000c spectrophotometer (Thermo Fisher Scientific, Waltham, MA, United States). The PCR product was *in vitro* transcribed using T7 RNA polymerase (Agilent Technologies, Santa Clara, CA, United States) for 6 h at 30°C, 500 rpm on a Thermomix Comfort (Eppendorf, Hamburg, Germany) and subsequently treated with DNaseI (NEB, Ipswich, MA, United States). tRNA was purified by guanidinium thiocyanate-phenol-chloroform extraction using TRIzol (Thermo Fisher Scientific, Waltham, MA, United States) and diluted to 100 µM using ultrapure water after RNA quantification using a Nanodop-2000c spectrophotometer (Thermo Fisher Scientific, Waltham, MA, United States). Folding of tRNA was achieved using the following PCR program: 120 s 80°C, 30 s 75°C, 30 s 70°C, 30 s 65°C, 30 s 60°C, 30 s 55°C, 30 s 50°C, 30 s 45°C, 30 s 40°C, 30 s 35°C, 300 s 25°C, 4°C. tRNA was snap frozen in liquid nitrogen and stored at −80°C. AzF was purchased from Bachem (Bubendorf, Switzerland).

### Cell-Free Protein Synthesis for Site-Specific ncAA Labeling

Cell-free protein synthesis for site-specific ncAA labeling was performed based on translationally active CHO lysates as described previously (Stech et al., 2021). In brief, cell-free reactions were composed of three different premixes (A, B, C). Premix A (10x) consisted of 300 mM HEPES-KOH (pH 7.6), 1,350 mM KOAc, 2.5 mM spermidine, 1 mM of each canonical amino acids except leucine and tyrosine (Merck, Darmstadt, Germany), 1 mM Tyr in KOH, 0.5 mM leucine, 0.33 mM ^14^C leucine, and 39 mM Mg(OAc)_2_. Premix B (2.5x) was prepared, as described previously (Thoring and Kubick, 2018), containing S7 nuclease-treated CHO lysate supplemented with 250 μg/ml creatine kinase (Roche, Basel, Switzerland) and 50 μg/ml bulk yeast tRNA (Roche, Basel, Switzerland) (f.c. 40% CHO lysate in the reaction). Premix C (5x) contained 100 mM creatine phosphate, 8.75 mM ATP, 1.5 mM CTP, 1.5 mM UTP, 1.5 mM GTP (Roche, Basel, Switzerland), and 1.65 mM m^7^G (ppp)G cap analog (Prof. Edward Darzynkiewicz, Warsaw University, Poland). On ice, premix A, premix B, and premix C were diluted to 1x concentrated solution in ultrapure water and supplemented with 3 U/μL (f.c.) T7 RNA polymerase (Agilent, Santa Clara, CA, United States) and plasmid (f.c. 5–10 nM). For subsequent qualitative and quantitative analysis by autoradiography and liquid scintillation counting, ^14^C-leucine (f.c. 100 μM) was added to cell-free reactions (specific radioactivity 133.34 dpm/pmol, Perkin Elmer, Waltham, MA, United States). For incorporation of ncAAs into proteins, TyrRS (f.c. 0.3–3 µM), tRNATyr_CUA_ (f.c. 0.5–5 µM), and AzF (f.c. 0.2–2 mM, Bachem, Bubendorf, Switzerland) were added to the reaction mixture. The reactions were carried out in triplicates at 30°C for 2–10 h without agitation in volumes between 1.5 and 50 µl in a 96-well plate in a CFX96 Touch qPCR cycler (Biorad, Hercules, CA, United States), and fluorescence was detected in the FAM channel (green, excitation: 450–490 nm, detection: 510–530 nm) and the Texas Red channel (red, excitation: 560–590 nm, detection: 610–650 nm).

### Endpoint Fluorescence Measurement and Fractionation of Cell-Free Reactions in SN1 and SN2

Generally, endpoint RFU measurements of the translation mix (TM), the SN1 fraction, and the SN2 fraction were carried out in a volume adjusted to 30 µl with phosphate-buffered saline (PBS, pH 7.4). For obtaining the supernatant of the translation mix, representing the soluble fraction of cell-free synthesized, non-translocated target proteins (SN1), triplicates of PBS-diluted cell-free reactions were pooled and transferred to 1.5 ml reaction tubes, centrifuged at 16,000 × g, 10 min, 4°C, and the SN1 supernatant was transferred to a new reaction tube. After removal of SN1, the microsomal pellet was washed with 50 µl of PBS, centrifuged, and the supernatant was discarded. The washed pellet was resuspended in PBS supplemented with 0.2% n-Dodecyl β-D-maltoside (DDM) (Iris Biotech, Marktredwitz, Germany) and rigorously agitated for 45 min at RT to release the translocated antibodies from the lumen of the microsomal vesicles. To obtain the soluble fraction of the microsome-translocated antibodies (SN2), the solutions were centrifuged, and the SN2 supernatant was transferred to the qPCR cycler for measurement of triplicates or to a new reaction tube for storage at −20°C after snap frozen in liquid nitrogen.

### Calculation of Readthrough Efficiency and Maximum Misincorporation Frequency

Relative readthrough efficiency (RRE) was calculated by the following formula: (RFU FAM/RFU TX RED)_AMB_/(RFU FAM/RFU TX RED)_WT_ in the presence of orthogonal components aaRS, tRNA, and ncAA. For calculation of the maximum misincorporation frequency (MMF), the RRE in the absence (-ortho) was divided by the RRE in the presence (+ortho) of orthogonal components aaRS, tRNA, and ncAA.

### Determination of Total Protein Yields by Liquid Scintillation Counting

Determination of total protein yields was performed in triplicates based on liquid scintillation as described previously (Stech et al., 2017). Following cell-free protein synthesis and fractionation into SN1 and SN2, aliquots were withdrawn from the solution, mixed with 3 ml trichloroacetic acid (TCA), and incubated in an 80°C water bath for 15 min, followed by incubation on ice for 30 min. In order to remove non-incorporated ^14^C-leucine, protein solutions were filtered using a vacuum filtration system (Hoefer, Holliston, MA, United States) and glass fiber filters (Machery-Nagel, Düren, Germany). Incorporation of ^14^C-leucine in cell-free synthesized proteins was measured by liquid scintillation counting using the HIDEX 600 SL (Hidex, Turku, Finland).

### Labeling of AzF-scFv With Fluorescent Dye (Staudinger Ligation)

An aliquot containing 30 fmol of cell-free synthesized scFv from the SN2 fraction was labeled with DyLight™ 650-Phosphine (f.c. 10 μM, Thermo Fisher Scientific, Waltham, MA, United States) by incubation at 25°C and 600 rpm protected from light for 90 min in a total volume of 15 μl filled up with ultrapure water.

### Sodium Dodecyl Sulfate–Polyacrylamide Gel Electrophoresis, Autoradiography, and In-Gel Fluorescence

Sodium dodecyl sulfate polyacrylamide gel electrophoresis (SDS-PAGE) was performed using 10% or 12% Tris-Glycine gels under reducing, denaturing conditions. In general, aliquots equaling 3 µl of undiluted SN1 or SN2 were filled up to 50 µl with ultrapure water on ice and 150 µl of ice cold acetone was added. For precipitation of proteins, reaction tubes were incubated on ice for 15 min, centrifuged (16,000 × g, 10 min, 4°C), and the supernatant was discarded. A total of 15 µl of 1x LDS buffer (Invitrogen, Carlsbad, CA, United States) supplemented with Dithiothreitol (f.c. 50 mM) was added to the dried precipitated protein pellet and incubated under rigorous shaking at room temperature for 15 min, followed by incubation at 70°C for 10 min and loading onto PAGE gels, which were run for 60 min at 150 V. The samples analyzed in in-gel fluorescence were not denatured. For autoradiography following electrophoresis, gels were stained with Coomassie Blue (SimplyBlue SafeStain, Thermo Fisher Scientific, Waltham, MA, United States). After staining, gels were dried on Whitman paper for 60–70 min at 70°C (Unigeldryer 3545D, Uniequip, Planegg, Germany), and radioactively labeled proteins were visualized using a phosphorimager system (Amersham Typhoon RGB Biomolecular Imager, GE Healthcare, Chicago, IL, United States). For in-gel fluorescence, gels were washed in dH_2_O after electrophoresis. The labeled proteins were visualized by using a phosphorimager system (excitation 633 nm, emission 670 nm, Amersham™ Typhoon™ RGB Biomolecular Imager, GE Healthcare, Chicago, IL, United States).

### Enzyme-Linked Immunosorbent Assay

The functional analysis of cell-free synthesized scFv was performed by Enzyme-linked Immunosorbent Assay (ELISA) in triplicates by overnight coating at 4°C of a 96-well microtiter plate (Corning, Wiesbaden, Germany) with 20 nM (f.c.) recombinant EGFR-His (ECD L25-S645, Acro Biosystems, Newark, DE, United States) in 50 µl of 0.1 M sodium carbonate buffer pH 9.6 per well. The next day, the plate was washed thrice with 200 µl of PBS-T (PBS containing 0.05% Tween-20), and the EGFR-coated wells and the empty wells serving as no antigen control (nAg) were blocked with 200 µl of PBS containing 2% BSA for 2–4 h on a rotary shaker. The washing step was repeated, and the EGFR-coated wells and nAg wells were incubated with 50 µl of cell-free synthesized scFv diluted in PBS containing 1% of BSA for 2 h at 300 rpm. For the ELISA binding curve with PNT^WT^ and RFP-PNT^WT^-GFP, a 10 point 1:2 serial dilution starting from 1 nM f.c. was prepared. For ELISA with RFP-PNT-GFP amber mutants, 100 pM of suppression product per well was used as determined from the FAM RFU signal and the standard curve. After another washing step, the wells were incubated with 50 µl of Strep-Tactin®-HRP (IBA, Göttingen, Germany) diluted 1:2,000 in PBS containing 1% BSA to bind the C-terminal TwinStrep-Tag of full-length antibodies. After washing thrice with PBS-T and an additional washing step with 200 µl of PBS, the wells were incubated with 100 µl of TMB substrate solution (Thermo Fisher Scientific, Waltham, MA, United States) diluted 1:2 in ultrapure H_2_O for 5–15 min. Color development was stopped by adding 100 µl of 0.5 M H_2_SO_4_, and absorbance was measured in a FLUOstar Omega plate reader (BMG Labtech, Ortenberg, Germany) at 450 nm (reference 620 nm). The A_450-620_ signal of empty nAg wells was subtracted from the A_450-620_ signal of target-coated wells to obtain a value indicating specific binding against block control.

## Data Availability

The original contributions presented in the study are included in the article/Supplementary Material, further inquiries can be directed to the corresponding author.

## Supplementary Material

Supplementary Figure S1 Autoradiograph and liquid scintillation quantification of cell-free synthesized reporter proteins in the SN1 fraction.

Supplementary Figure S2 Ratio of GFP and RFP signal of reporter proteins according to sample dilution and volume.

Supplementary Figure S3 RNase addition during cell-free translation of fluorescent reporter proteins.

Supplementary Figure S4 Autoradiograph of scFv with and without fluorescent reporter synthesized in SN1 and SN2 fraction.

Supplementary Figure S5 Correlation of liquid scintillation quantification with fluorescence.

Supplementary Figure S6 Quantification of fluorescence for amber suppression of reporter RFP-X-GFP with varying concentrations for orthogonal components and SDS-PAGE autoradiography.

Supplementary Figure S7 Fluorescence of RFP-scFv-GFP amber mutants and reporter proteins RFP-X-GFP and RFP-Y-GFP.

Supplementary Figure S8 Plasmid sequences.

Supplementary Table S1 Primer sequences.

Supplementary Table S2 Primer combinations.

## Abbreviations

aaRS, aminoacyl tRNA synthetase; ADC, antibody-drug conjugate; AzF, p-azido-l-phenylalanine; cAA, canonical amino acid; CFPS, cell-free protein synthesis; EGFR, epidermal growth factor receptor; ER, endoplasmic reticulum; IRES, internal ribosomal entry site; MS, mass spectrometry; MMF, maximum misincorporation frequency; ncAA, noncanonical amino acid; NTC, no-template control; OTS, orthogonal translation system; PNT, panitumumab; RRE, relative readthrough efficiency; scFv, single-chain variable fragment.
