# Supplementary figures and images for: A CHO-Based Cell-Free Dual Fluorescence Reporter System for the Straightforward Assessment of Amber Suppression and scFv Functionality
Source: Front Bioeng Biotechnol. 2022 Apr 29;10:873906. doi: 10.3389/fbioe.2022.873906 (PMC9098822; doi:10.3389/fbioe.2022.873906)

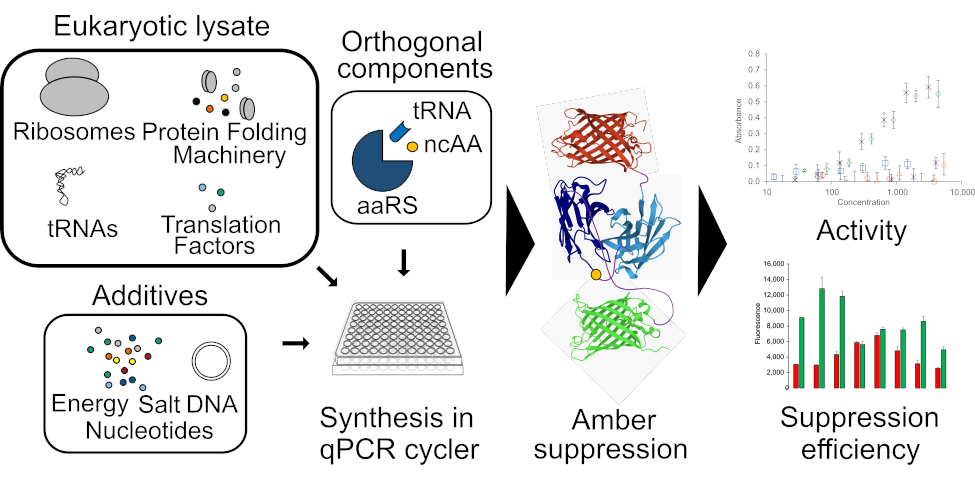

Supplement: Supplementary file 1 [file Image1.TIF]
