# Supplementary material for: A CHO-Based Cell-Free Dual Fluorescence Reporter System for the Straightforward Assessment of Amber Suppression and scFv Functionality
Source: Front Bioeng Biotechnol. 2022 Apr 29;10:873906. doi: 10.3389/fbioe.2022.873906 (PMC9098822; doi:10.3389/fbioe.2022.873906)
Supplement: Supplementary file 2 [file DataSheet1.docx]

Supplementary Material


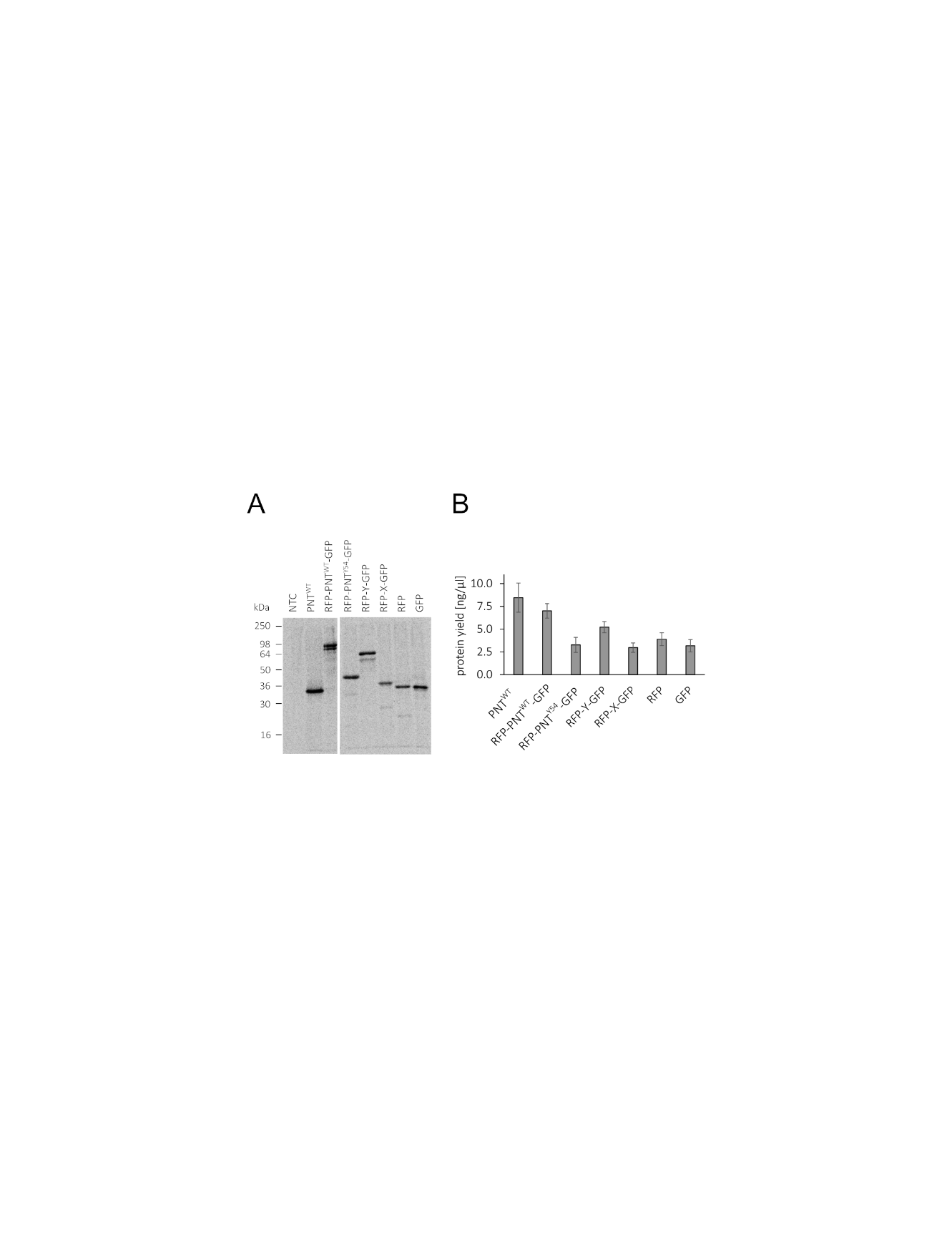


**Supplementary Figure S1: Qualitative and quantitative analysis of cell-free synthesized reporter proteins in the supernatant fraction (SN1). (A)** Autoradiograph of 12 % Tris-Glycine SDS-PAGE (PNT^WT^: 32.5 kDa; RFP-PNT^WT^-GFP: 87.9 kDa; RFP-PNT^Y54^-GFP: 36.0 kDa; RFP-Y-GFP: 58.5 kDa; RFP-X-GFP: 30.1 kDa, RFP: 28.2 kDa; GFP: 29.6 kDa). **(B)** Protein yield determined by liquid scintillation counting. Mean values of triplicates are plotted with error bars representing their standard deviation.

**
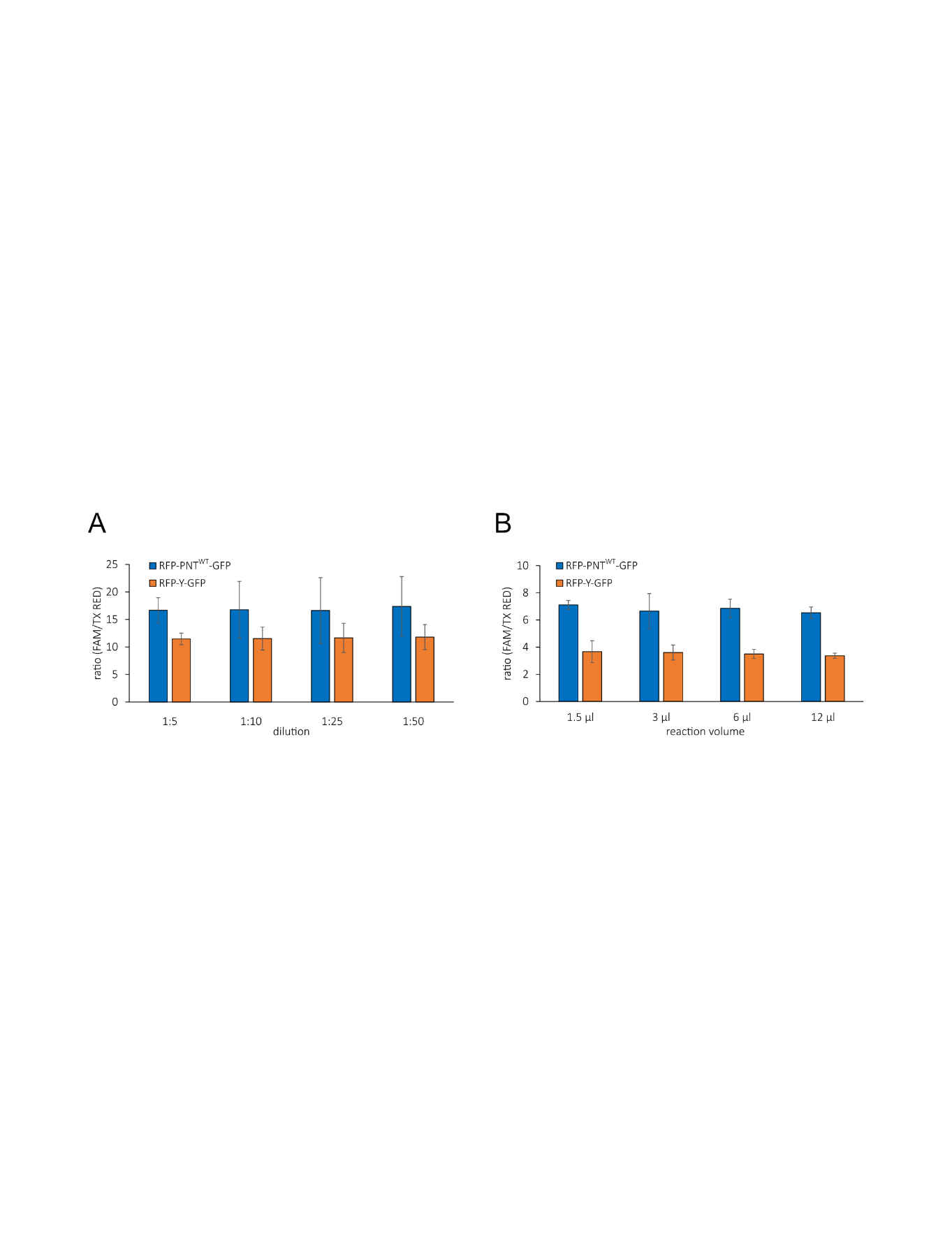
**

**Supplementary Figure S2: Ratio of GFP and RFP signal of reporter proteins to assess robustness of fluorescence measurements. (A)** For different dilutions with PBS in a two hour reaction and **(B)** in different reaction volumes in a three hour reaction. Mean values of triplicates were background substracted and their ratios were plotted with error bars representing their standard deviation.


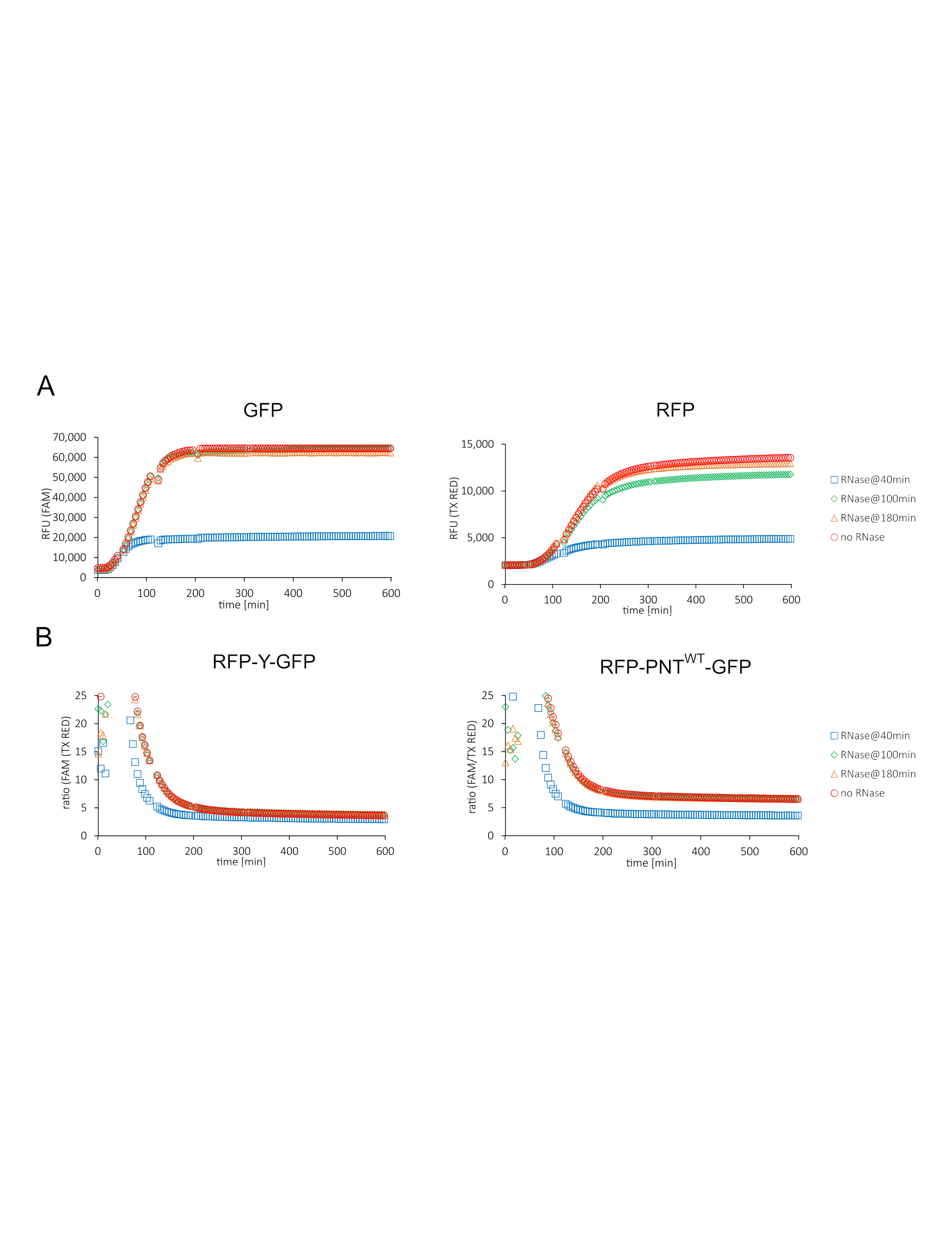


**Supplementary Figure S3: RNase addition during cell-free translation of fluorescent reporter proteins. (A)** Fluorescence over time for GFP in FAM channel (left) and RFP in the Texas Red channel (right). **(B)** Ratio of RFU (FAM/TX RED) for RFP-Y-GFP (left) and RFP.PNT^WT^-GFP (right).

**
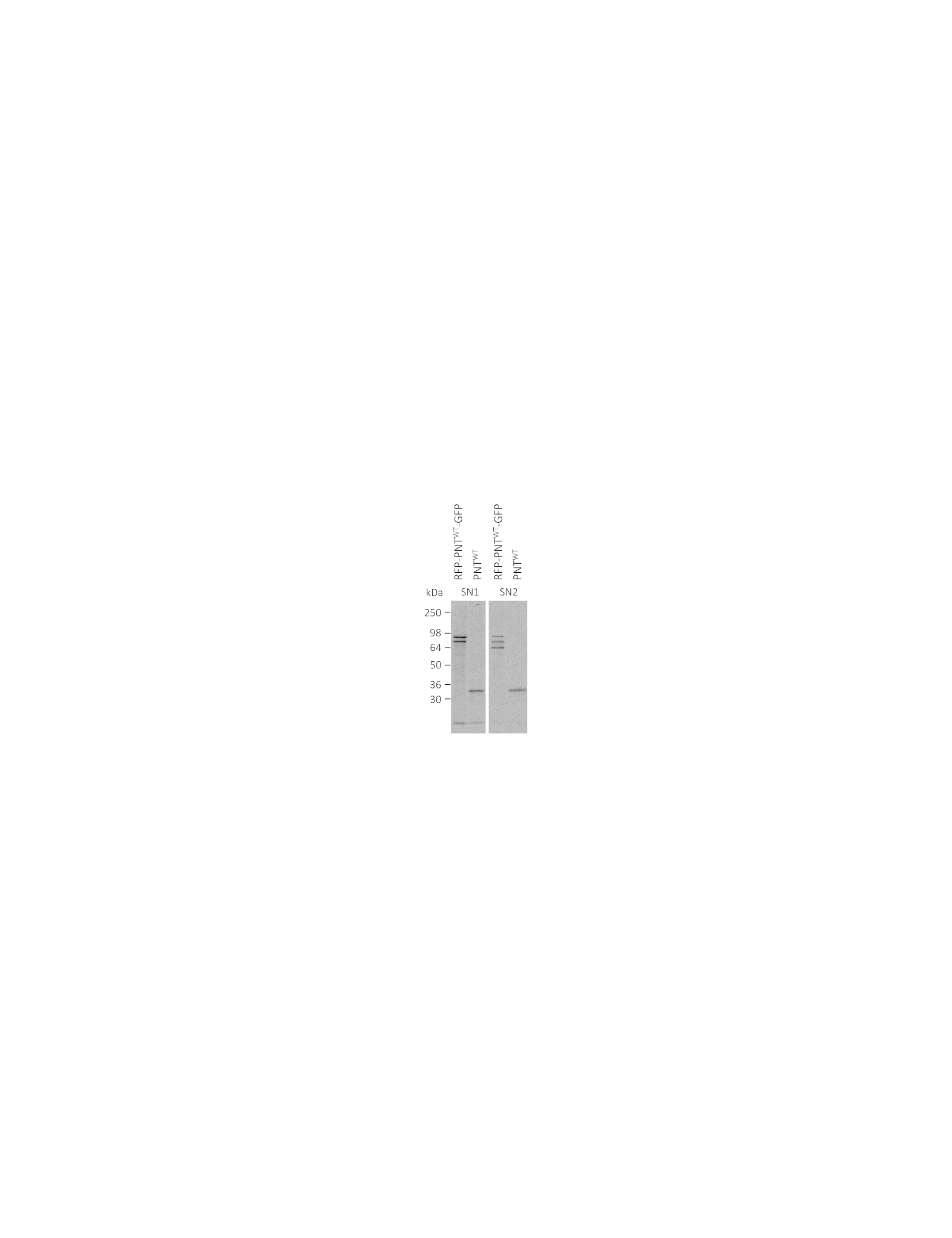
**

**Supplementary Figure S4: Cell-free expression of panitumumab scFv with and without fluorescent reporter proteins**. Autoradiograph of 10 % Tris-Glycine SDS-PAGE visualizing the soluble fraction of the translation mix (SN1) and the soluble fraction extracted from ER-derived microsomes (SN2).


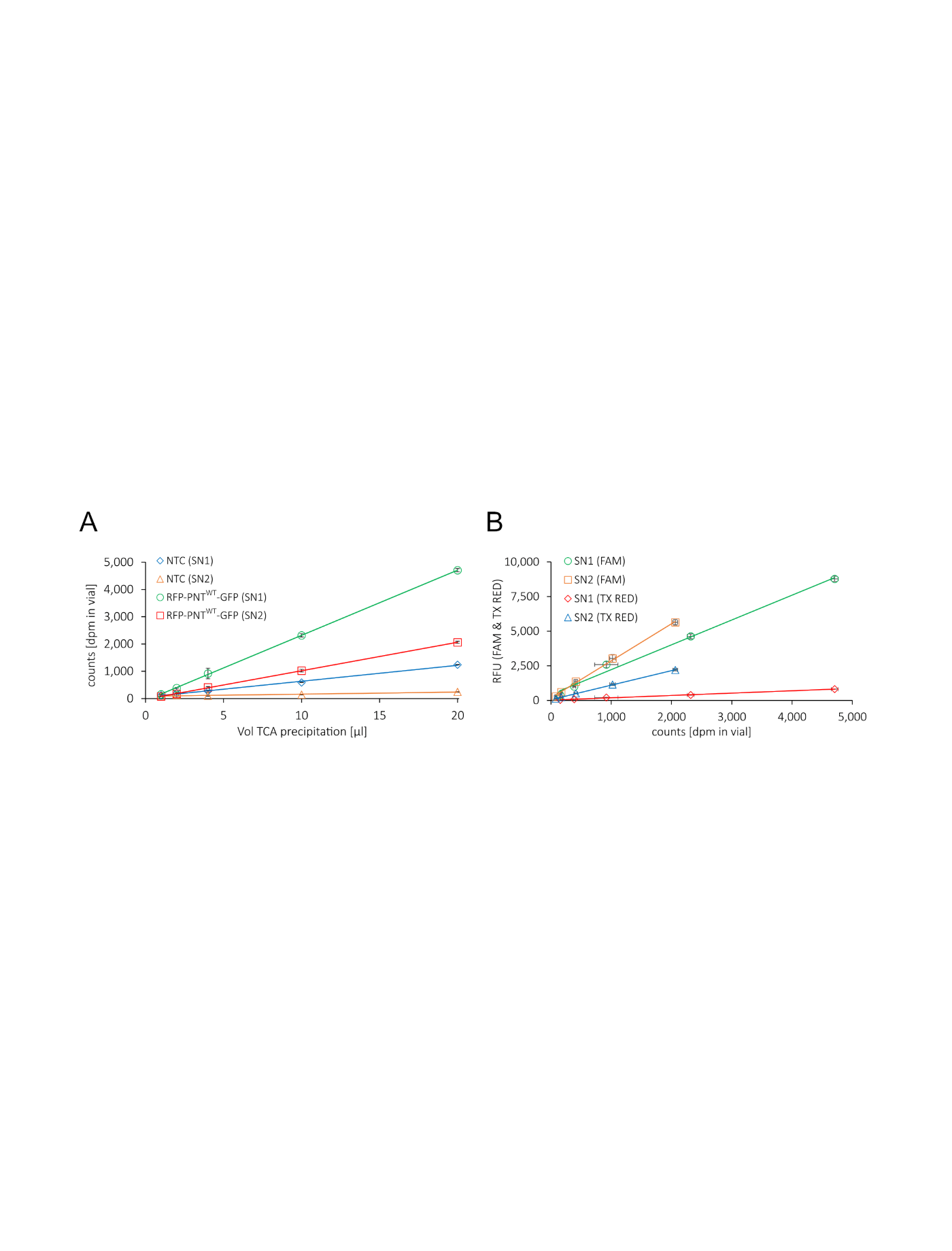


**Supplementary Figure S5: Correlation of liquid scintillation quantification with fluorescence. (A)** Radioactive counts in relation to volume used for liquid scintillation quantification for NTC and RFP-PNT^WT^-GFP in fraction SN1 and SN2 **(B)** Corresponding background substracted fluorescence of RFP-PNT^WT^-GFP in relation to radioactive counts in the FAM and Texas Red channel for fractions SN1 and SN2. Mean values of triplicates are plotted with error bars representing their standard deviation.


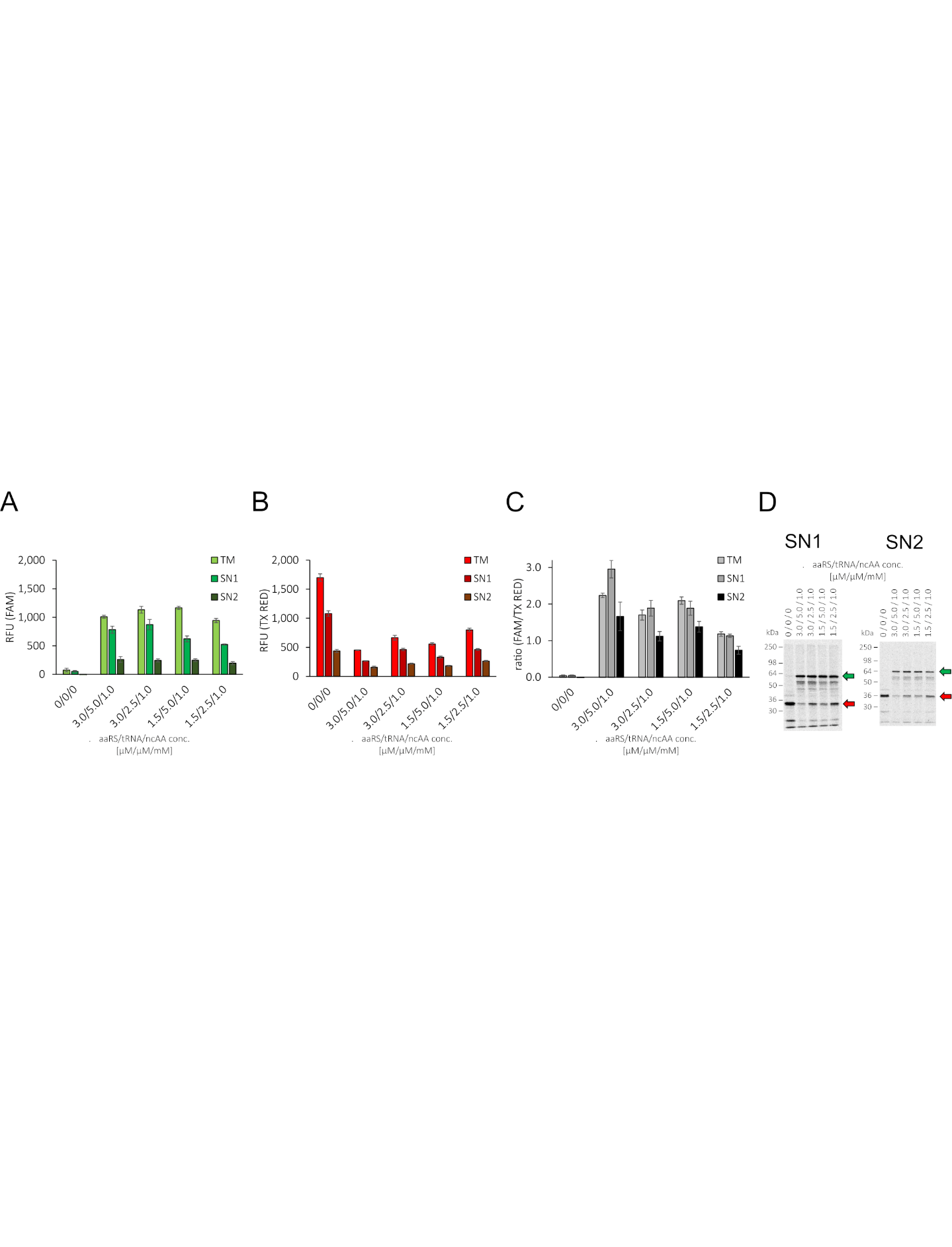


**Supplementary Figure S6: Quantification of fluorescence for amber suppression of reporter RFP-X-GFP with varying concentrations for orthogonal components and SDS-PAGE autoradiography.** Background substracted fluorescence signals of the reporter in the cell-free translation mix (TM) and the supernatant fractions SN1 and SN2 **(A)** in the FAM channel, **(B)** the Texas Red channel and **(C)** their proportion as FAM/TX RED ratio. **(D)** Autoradiograph of SDS-PAGE of the SN1 and the SN2 fraction. The green arrow depicts the full-length product and the red arrow the termination product. Mean values of triplicates are plotted with error bars representing their standard deviation.


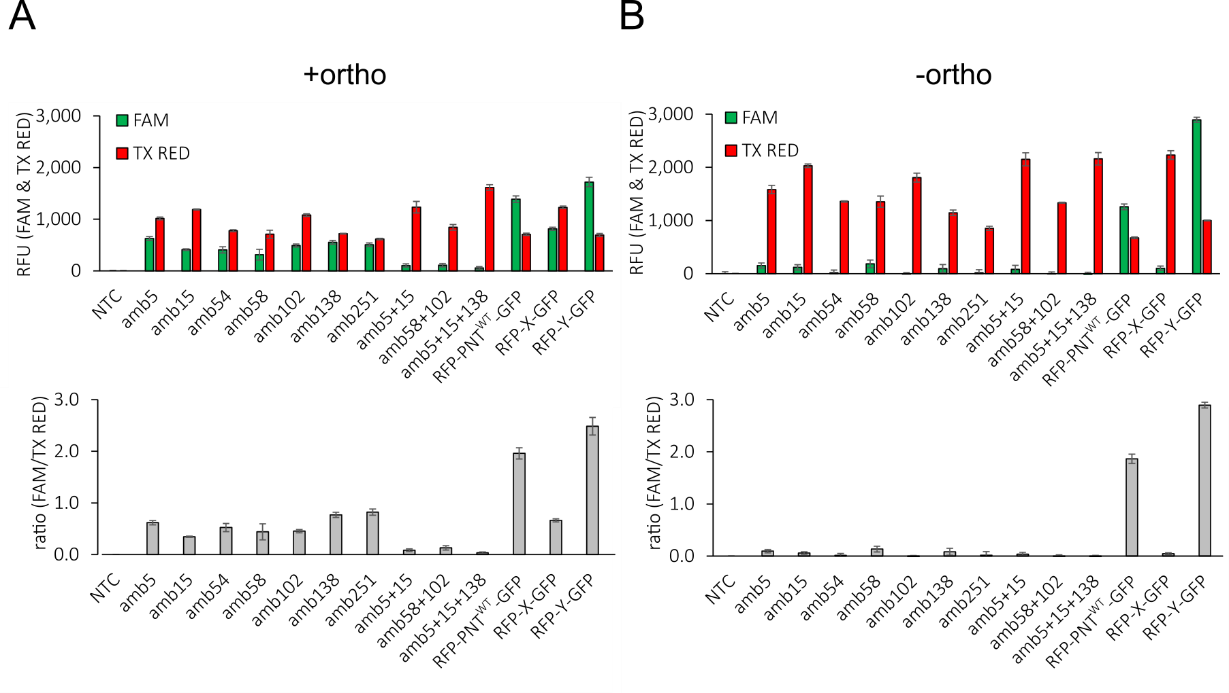


**Supplementary Figure S7: Fluorescence of RFP-PNT-GFP amber mutants, RFP-PNT^WT^-GFP, RFP-X-GFP and RFP-Y-GFP in the FAM channel, the Texas Red channel and resulting ratio (FAM/TX RED) (A)** with orthogonal components concentration of 1.5 µM aaRS, 2.5 µM tRNA and 1 mM ncAA and **(B)** without orthogonal components. Mean values of triplicates were fluorescence background substracted and are plotted with error bars representing their standard deviation.

**>pUC57-1.8k_NCM+D_mRFP1-Y-sfGFP**

GATATGATATACGTACGATAGGCTAGCTAATACGACTCACTATAGGGAGACCACAACGGTTTCCCTCTAGAAATAATTTTGTTTAACTTTAAGAAGGAGATAAACAAAAGCAAAAATGTGATCTTGCTTGTAAATACAATTTTGAGAGGTTAATAAATTACAAGTAGTGCTATTTTTGTATTTAGGTTAGCTATTTAGCTTTACGTTCCAGGATGCCTAGTGGCAGCCCCACAATATCCAGGAAGCCCTCTCTGCGGTTTTTCAGATTAGGTAGTCGAAAAACCTAAGAAATTTACCT**GCT**AAATTCTTAGTCAACGTTGCCCTTGTTTTTATGGTC**GTATAC**ATTTCTTACATCTATGCGGACATGGCCTCTTCCGAGGACGTGATCAAAGAATTTATGCGGTTCAAAGTGCGGATGGAAGGCTCCGTGAACGGCCACGAGTTTGAGATCGAAGGCGAAGGCGAGGGCAGACCTTATGAGGGCACACAGACCGCCAAGCTGAAAGTGACCAAAGGCGGCCCTCTGCCTTTTGCCTGGGATATCCTGTCTCCTCAGTTCCAGTACGGCTCCAAGGCCTACGTGAAGCACCCTGCTGACATCCCCGACTACCTGAAGCTGTCTTTCCCAGAGGGCTTCAAGTGGGAGAGAGTGATGAACTTCGAGGACGGCGGCGTGGTCACCGTGACTCAAGATAGCTCTCTGCAGGACGGCGAGTTCATCTACAAAGTGAAGCTGCGGGGCACCAACTTTCCCTCTGATGGCCCCGTGATGCAGAAAAAGACGATGGGCTGGGAAGCCTCCACCGAGAGAATGTACCCCGAGGATGGCGCACTcAAGGGCGAGATAAAGATGCGGCTGAAGCTGAAGGATGGCGGCCACTACGACGCCGAAGTGAAAACCACCTACATGGCCAAGAAACCCGTGCAGCTGCCTGGCGCCTACAAGACCGATATCAAGCTGGACATCACCAGCCACAACGAGGACTACACCATCGTGGAACAGTACGAGAGAGCCGAAGGCAGACACTCCACCGGCGCTAAAGAATCCGGCTCCGTGTCCTCTGAACAGCTGGCCCAGTTCAGATCCCTGGACGGCGAATACGCATCCGAGGGCAAGTCCTCTGGCTCTGGATCAGAGTCCAAGTCCACAATGTCCAAGGGCGAAGAACTGTTCACCGGCGTGGTGCCCATTCTGGTGGAACTGGATGGGGATGTGAATGGCCACAAGTTCTCCGTCCGCGGAGAAGGCGAAGGGGATGCTACAAACGGCAAGCTGACCCTGAAGTTTATCTGCACCACCGGAAAGCTGCCCGTGCCTTGGCCTACACTGGTCACCACACTGACCTACGGCGTGCAGTGCTTCAGCAGATACCCCGACCATATGAAGCGGCACGACTTCTTCAAGTCCGCCATGCCTGAGGGCTACGTGCAAGAGCGGACCATCTCCTTCAAGGACGACGGCACCTATAAGACCAGAGCTGAAGTGAAGTTCGAGGGCGACACCCTGGTCAACCGGATCGAGCTGAAGGGCATCGATTTCAAAGAGGACGGCAACATCCTGGGCCACAAGCTCGAGTACAACTTCAACTCCCACAACGTGTACATCACCGCCGACAAGCAGAAGAACGGCATCAAGGCCAACTTCAAGATCCGGCACAACGTGGAAGATGGCTCTGTGCAGCTGGCCGACCACTACCAGCAGAACACCCCTATCGGAGATGGCCCAGTGCTGCTGCCTGACAACCACTACCTGTCTACCCAGTCCGTGCTGAGCAAGGACCCTAACGAGAAGCGGGACCACATGGTGCTGCTGGAATTTGTGACCGCCGCTGGCATCACCCACGGCATGGATGAGCTGTACAAG**TAATAACTAACTAACCAAg**ATCTgTACCCCTTggggCCTCTAAACgggTCTTgAggggTTTTTTggATCCgAATTCACCggTGATATCATATCACATGTGAGCAAAAGGCCAGCAAAAGGCCAGGAACCGTAAAAAGGCCGCGTTGCTGGCGTTTTTCCATAGGCTCCGCCCCCCTGACGAGCATCACAAAAATCGACGCTCAAGTCAGAGGTGGCGAAACCCGACAGGACTATAAAGATACCAGGCGTTTCCCCCTGGAAGCTCCCTCGTGCGCTCTCCTGTTCCGACCCTGCCGCTTACCGGATACCTGTCCGCCTTTCTCCCTTCGGGAAGCGTGGCGCTTTCTCATAGCTCACGCTGTAGGTATCTCAGTTCGGTGTAGGTCGTTCGCTCCAAGCTGGGCTGTGTGCACGAACCCCCCGTTCAGCCCGACCGCTGCGCCTTATCCGGTAACTATCGTCTTGAGTCCAACCCGGTAAGACACGACTTATCGCCACTGGCAGCAGCCACTGGTAACAGGATTAGCAGAGCGAGGTATGTAGGCGGTGCTACAGAGTTCTTGAAGTGGTGGCCTAACTACGGCTACACTAGAAGAACAGTATTTGGTATCTGCGCTCTGCTGAAGCCAGTTACCTTCGGAAAAAGAGTTGGTAGCTCTTGATCCGGCAAACAAACCACCGCTGGTAGCGGTGGTTTTTTTGTTTGCAAGCAGCAGATTACGCGCAGAAAAAAAGGATCTCAAGAAGATCCTTTGATCTTTTCTACGGGGTCTGACGCTCAGTGGAACGAAAACTCACGTTAAGGGATTTTGGTCATGAGATTATCAAAAAGGATCTTCACCTAGATCCTTTTAAATTAAAAATGAAGTTTTAAATCAATCTAAAGTATATATGAGTAAACTTGGTCTGACAGTTACCAATGCTTAATCAGTGAGGCACCTATCTCAGCGATCTGTCTATTTCGTTCATCCATAGTTGCCTGACTCCCCGTCGTGTAGATAACTACGATACGGGAGGGCTTACCATCTGGCCCCAGTGCTGCAATGATACCGCGAGACCCACGCTCACCGGCTCCAGATTTATCAGCAATAAACCAGCCAGCCGGAAGGGCCGAGCGCAGAAGTGGTCCTGCAACTTTATCCGCCTCCATCCAGTCTATTAATTGTTGCCGGGAAGCTAGAGTAAGTAGTTCGCCAGTTAATAGTTTGCGCAACGTTGTTGCCATTGCTACAGGCATCGTGGTGTCACGCTCGTCGTTTGGTATGGCTTCATTCAGCTCCGGTTCCCAACGATCAAGGCGAGTTACATGATCCCCCATGTTGTGCAAAAAAGCGGTTAGCTCCTTCGGTCCTCCGATCGTTGTCAGAAGTAAGTTGGCCGCAGTGTTATCACTCATGGTTATGGCAGCACTGCATAATTCTCTTACTGTCATGCCATCCGTAAGATGCTTTTCTGTGACTGGTGAGTACTCAACCAAGTCATTCTGAGAATAGTGTATGCGGCGACCGAGTTGCTCTTGCCCGGCGTCAATACGGGATAATACCGCGCCACATAGCAGAACTTTAAAAGTGCTCATCATTGGAAAACGTTCTTCGGGGCGAAAACTCTCAAGGATCTTACCGCTGTTGAGATCCAGTTCGATGTAACCCACTCGTGCACCCAACTGATCTTCAGCATCTTTTACTTTCACCAGCGTTTCTGGGTGAGCAAAAACAGGAAGGCAAAATGCCGCAAAAAAGGGAATAAGGGCGACACGGAAATGTTGAATACTCATACTCTTCCTTTTTCAATATTATTGAAGCATTTATCAGGGTTATTGTCTCATGAGCGGATACATATTTGAATGTATTTAGAAAAATAAACAAATAGGGGTTCCGCGCACATTTCCCCGAAAAGTGCCACCTGACGTC

**>pUC57-1.8k_NCM+D_mRFP1-PNT.WT-sfGFP**

GATATGATATACGTACGATAGGCTAGCTAATACGACTCACTATAGGGAGACCACAACGGTTTCCCTCTAGAAATAATTTTGTTTAACTTTAAGAAGGAGATAAACAAAAGCAAAAATGTGATCTTGCTTGTAAATACAATTTTGAGAGGTTAATAAATTACAAGTAGTGCTATTTTTGTATTTAGGTTAGCTATTTAGCTTTACGTTCCAGGATGCCTAGTGGCAGCCCCACAATATCCAGGAAGCCCTCTCTGCGGTTTTTCAGATTAGGTAGTCGAAAAACCTAAGAAATTTACCTGCTAAATTCTTAGTCAACGTTGCCCTTGTTTTTATGGTCGTATACATTTCTTACATCTATGCGGACATGGCCTCTTCCGAGGACGTGATCAAAGAATTTATGCGGTTCAAAGTGCGGATGGAAGGCTCCGTGAACGGCCACGAGTTTGAGATCGAAGGCGAAGGCGAGGGCAGACCTTATGAGGGCACACAGACCGCCAAGCTGAAAGTGACCAAAGGCGGCCCTCTGCCTTTTGCCTGGGATATCCTGTCTCCTCAGTTCCAGTACGGCTCCAAGGCCTACGTGAAGCACCCTGCTGACATCCCCGACTACCTGAAGCTGTCTTTCCCAGAGGGCTTCAAGTGGGAGAGAGTGATGAACTTCGAGGACGGCGGCGTGGTCACCGTGACTCAAGATAGCTCTCTGCAGGACGGCGAGTTCATCTACAAAGTGAAGCTGCGGGGCACCAACTTTCCCTCTGATGGCCCCGTGATGCAGAAAAAGACGATGGGCTGGGAAGCCTCCACCGAGAGAATGTACCCCGAGGATGGCGCACTcAAGGGCGAGATAAAGATGCGGCTGAAGCTGAAGGATGGCGGCCACTACGACGCCGAAGTGAAAACCACCTACATGGCCAAGAAACCCGTGCAGCTGCCTGGCGCCTACAAGACCGATATCAAGCTGGACATCACCAGCCACAACGAGGACTACACCATCGTGGAACAGTACGAGAGAGCCGAAGGCAGACACTCCACCGGCGCTAAAGAATCCGGCTCCGTGTCCTCTGAACAGCTGGCCCAGTTCAGATCCCTGGACTCCATGGGAGGTTCACAGGTTCAGCTGCAAGAGTCTGGACCTGGCCTGGTCAAGCCTTCCGAGACACTGTCTCTGACCTGCACCGTGTCTGGCGGCTCTGTGTCCTCTGGCGATTACTATTGGACCTGGATTCGGCAGTCCCCTGGCAAAGGACTGGAATGGATCGGCCACATCTACTACTCCGGCAACACCAACTACAACCCCAGCCTGAAGTCCCGGCTGACCATCTCCATCGACACCAGCAAGACCCAGTTCTCCCTGAAGCTGTCCTCTGTGACCGCCGCTGATACCGCCATCTACTATTGCGTGCGGGACAGAGTGACCGGCGCCTTTGATATTTGGGGCCAGGGCACAATGGTCACCGTTTCTAGCGGTGGCGGAGGTTCTGGCGGTGGAGGTTCTGGCGGAGGCGGAAGTGGCGGTGGAGGTAGTGATATCCAGATGACCCAGTCTCCTTCCAGCCTGTCTGCCTCTGTGGGCGATAGAGTGACCATCACCTGTCAGGCCAGCCAGGACATCTCCAACTACCTGAACTGGTATCAGCAGAAGCCCGGCAAGGCCCCTAAGCTGCTGATCTACGATGCCTCCAACCTGGAAACCGGCGTGCCCTCTAGATTCTCCGGCTCTGGCTCTGGCACCGACTTTACCTTTACAATCTCCAGCCTGCAGCCTGAGGATATCGCCACCTACTTTTGCCAGCACTTCGATCATCTGCCCCTGGCCTTTGGCGGAGGCACCAAGGTGGAAATCAAGGCGGCCGCAGGAGGAAGTGGCGGTTCCAGCGCATGGTCCCATCCTCAGTTCGAGAAAGGCGGAGGATCTGGCGGAGGTAGCGGTGGATCTAGTGCTTGGAGCCACCCCCAGTTTGAAAAGGAGGGCAAGTCCTCTGGCTCTGGATCAGAGTCCAAGTCCACAATGTCCAAGGGCGAAGAACTGTTCACCGGCGTGGTGCCCATTCTGGTGGAACTGGATGGGGATGTGAATGGCCACAAGTTCTCCGTCCGCGGAGAAGGCGAAGGGGATGCTACAAACGGCAAGCTGACCCTGAAGTTTATCTGCACCACCGGAAAGCTGCCCGTGCCTTGGCCTACACTGGTCACCACACTGACCTACGGCGTGCAGTGCTTCAGCAGATACCCCGACCATATGAAGCGGCACGACTTCTTCAAGTCCGCCATGCCTGAGGGCTACGTGCAAGAGCGGACCATCTCCTTCAAGGACGACGGCACCTATAAGACCAGAGCTGAAGTGAAGTTCGAGGGCGACACCCTGGTCAACCGGATCGAGCTGAAGGGCATCGATTTCAAAGAGGACGGCAACATCCTGGGCCACAAGCTCGAGTACAACTTCAACTCCCACAACGTGTACATCACCGCCGACAAGCAGAAGAACGGCATCAAGGCCAACTTCAAGATCCGGCACAACGTGGAAGATGGCTCTGTGCAGCTGGCCGACCACTACCAGCAGAACACCCCTATCGGAGATGGCCCAGTGCTGCTGCCTGACAACCACTACCTGTCTACCCAGTCCGTGCTGAGCAAGGACCCTAACGAGAAGCGGGACCACATGGTGCTGCTGGAATTTGTGACCGCCGCTGGCATCACCCACGGCATGGATGAGCTGTACAAGTAATAACTAACTAACCAAgATCTgTACCCCTTggggCCTCTAAACgggTCTTgAggggTTTTTTggATCCgAATTCACCggTGATATCATATCACATGTGAGCAAAAGGCCAGCAAAAGGCCAGGAACCGTAAAAAGGCCGCGTTGCTGGCGTTTTTCCATAGGCTCCGCCCCCCTGACGAGCATCACAAAAATCGACGCTCAAGTCAGAGGTGGCGAAACCCGACAGGACTATAAAGATACCAGGCGTTTCCCCCTGGAAGCTCCCTCGTGCGCTCTCCTGTTCCGACCCTGCCGCTTACCGGATACCTGTCCGCCTTTCTCCCTTCGGGAAGCGTGGCGCTTTCTCATAGCTCACGCTGTAGGTATCTCAGTTCGGTGTAGGTCGTTCGCTCCAAGCTGGGCTGTGTGCACGAACCCCCCGTTCAGCCCGACCGCTGCGCCTTATCCGGTAACTATCGTCTTGAGTCCAACCCGGTAAGACACGACTTATCGCCACTGGCAGCAGCCACTGGTAACAGGATTAGCAGAGCGAGGTATGTAGGCGGTGCTACAGAGTTCTTGAAGTGGTGGCCTAACTACGGCTACACTAGAAGAACAGTATTTGGTATCTGCGCTCTGCTGAAGCCAGTTACCTTCGGAAAAAGAGTTGGTAGCTCTTGATCCGGCAAACAAACCACCGCTGGTAGCGGTGGTTTTTTTGTTTGCAAGCAGCAGATTACGCGCAGAAAAAAAGGATCTCAAGAAGATCCTTTGATCTTTTCTACGGGGTCTGACGCTCAGTGGAACGAAAACTCACGTTAAGGGATTTTGGTCATGAGATTATCAAAAAGGATCTTCACCTAGATCCTTTTAAATTAAAAATGAAGTTTTAAATCAATCTAAAGTATATATGAGTAAACTTGGTCTGACAGTTACCAATGCTTAATCAGTGAGGCACCTATCTCAGCGATCTGTCTATTTCGTTCATCCATAGTTGCCTGACTCCCCGTCGTGTAGATAACTACGATACGGGAGGGCTTACCATCTGGCCCCAGTGCTGCAATGATACCGCGAGACCCACGCTCACCGGCTCCAGATTTATCAGCAATAAACCAGCCAGCCGGAAGGGCCGAGCGCAGAAGTGGTCCTGCAACTTTATCCGCCTCCATCCAGTCTATTAATTGTTGCCGGGAAGCTAGAGTAAGTAGTTCGCCAGTTAATAGTTTGCGCAACGTTGTTGCCATTGCTACAGGCATCGTGGTGTCACGCTCGTCGTTTGGTATGGCTTCATTCAGCTCCGGTTCCCAACGATCAAGGCGAGTTACATGATCCCCCATGTTGTGCAAAAAAGCGGTTAGCTCCTTCGGTCCTCCGATCGTTGTCAGAAGTAAGTTGGCCGCAGTGTTATCACTCATGGTTATGGCAGCACTGCATAATTCTCTTACTGTCATGCCATCCGTAAGATGCTTTTCTGTGACTGGTGAGTACTCAACCAAGTCATTCTGAGAATAGTGTATGCGGCGACCGAGTTGCTCTTGCCCGGCGTCAATACGGGATAATACCGCGCCACATAGCAGAACTTTAAAAGTGCTCATCATTGGAAAACGTTCTTCGGGGCGAAAACTCTCAAGGATCTTACCGCTGTTGAGATCCAGTTCGATGTAACCCACTCGTGCACCCAACTGATCTTCAGCATCTTTTACTTTCACCAGCGTTTCTGGGTGAGCAAAAACAGGAAGGCAAAATGCCGCAAAAAAGGGAATAAGGGCGACACGGAAATGTTGAATACTCATACTCTTCCTTTTTCAATATTATTGAAGCATTTATCAGGGTTATTGTCTCATGAGCGGATACATATTTGAATGTATTTAGAAAAATAAACAAATAGGGGTTCCGCGCACATTTCCCCGAAAAGTGCCACCTGACGTC

**Annotation**

Restriction sites: NheI, NcoI, NotI, BglII, BamHi, EcoRI, AgeI

Regulatory elements: T7 promotor, IRES, stop codon, T7 terminator

Coding sequence: Mellitin signal peptide, mRFP1, Linker 1, V_H_ region, V_H_-V_L_ linker, V_L_ region, Twin Strep tag, Linker 2, sfGFP

**Supplementary Figure S8: DNA Sequence of RFP-Y-GFP and RFP-PNT^WT^-GFP in vector pUC57-1.8k.** Codons that are replaced with TAG for amb mutants are marked black.

**Supplementary Table S1: Primer to generate DNA template for tRNA in vitro transcription and DNA fragments for Gibson assembly.**

| # | Name | Sequence (5‘ -> 3‘) | Description | Position |
| --- | --- | --- | --- | --- |
| A01 | t_CUA_TyrEc_F | CGAGCTCGCCCACCGGAATTC | Forward primer amplifying tRNA sequence | 5’ |
| A02 | t_CUA_TyrEc_R | TGGTGGTGGGGGAAGGATTCG | Reverse primer amplifying tRNA sequence | 3’ |
| C01 | N0-T7p_F | GCTAATACGACTCACTATAGGG | Forward primer amplyfing 5’ sequence of all Gibson fragments | 5’ UTR |
| C02 | N0-T7p_R | CCCTATAGTGAGTCGTATTAGC | Reverse primer amplyfing pUC57-1.8k vector backbone Gibson fragment | 5’ UTR |
| C03 | C0-BglII_F | CTAACTAACCAAGATCTGTACC | Forward primer amplyfing pUC57-1.8k vector backbone Gibson fragment | 3’ UTR |
| C04 | C0-BglII_R | GGTACAGATCTTGGTTAGTTAG | Reverse primer amplyfing 3‘ sequence of all Gibson framgents | 3’ UTR |
| C05 | GS-G2amb-long_F | GGATAGAGTGGCGGTTCCAGC | Forward primer amplyfing PNT-G251amb scFv mutant | Strep tag Linker |
| C06 | GS-G2amb-long_R | GCTGGAACCGCCACTCTATCC | Reverse primer amplyfing PNT-G251amb scFv mutant | Strep tag Linker |
| C07 | PNT-Q005amb_F | TCACAGGTTCAGCTGTAGGAGTCTGGACCTGGCCTGGTCAAGC | Forward primer amplyfing PNT-Q5amb scFv mutant | FR-H1 |
| C08 | PNT-Q005amb_R | GCTTGACCAGGCCAGGTCCAGACTCCTACAGCTGAACCTGTGA | Reverse primer amplyfing PNT-Q5amb scFv mutant | FR-H1 |
| C09 | PNT-S015amb_F | GTCTGGACCTGGCCTGGTCAAGCCTTAGGAGACACTGTCTCTG | Forward primer amplyfing PNT-S15amb scFv mutant | FR-H1 |
| C10 | PNT-S015amb_R | CAGAGACAGTGTCTCCTAAGGCTTGACCAGGCCAGGTCCAGAC | Reverse primer amplyfing PNT-S15amb scFv mutant | FR-H1 |
| C11 | PNT-Y054amb_F | TGGATCGGCCACATCTAGTACTCCGGCAACACC | Forward primer amplyfing PNT-Y54amb scFv mutant | CDR-H2 |
| C12 | PNT-Y054amb_R | GGTGTTGCCGGAGTACTAGATGTGGCCGATCCA | Reverse primer amplyfing PNT-Y54amb scFv mutant | CDR-H2 |
| C13 | PNT-N058amb_F | ATCTACTACTCCGGCTAGACCAACTACAACCCC | Forward primer amplyfing PNT-N58amb scFv mutant | CDR-H2 |
| C14 | PNT-N058amb_R | GGGGTTGTAGTTGGTCTAGCCGGAGTAGTAGAT | Reverse primer amplyfing PNT-N58amb scFv mutant | CDR-H2 |
| C15 | PNT-V102amb_F | TGCGTGCGGGACAGATAGACCGGCGCCTTTGAT | Forward primer amplyfing PNT-V102amb scFv mutant | CDR-H3 |
| C16 | PNT-V102amb_R | ATCAAAGGCGCCGGTCTATCTGTCCCGCACGCA | Reverse primer amplyfing PNT-V102amb scFv mutant | CDR-H3 |
| C17 | PNT-G138amb_F | GGAAGTGGCGGTGGATAGAGTGATATCCAGATG | Forward primer amplyfing PNT-G138amb scFv mutant | V_H_-V_L_ Linker |
| C18 | PNT-G138amb_R | CATCTGGATATCACTCTATCCACCGCCACTTCC | Reverse primer amplyfing PNT-G138amb scFv mutant | V_H_-V_L_ Linker |
| D01 | Bird1_R | GTCCAGGGATCTGAACTGG | Reverse primer amplyfing mRFP1-backbone-sfGFP | Linker downstream of mRFP |
| D02 | Bird2_F | GAGGGCAAGTCCTCTGG | Forward primer amplyfing mRFP1-backbone-sfGFP | Linker upstream of sfGFP |
| D03 | Bird1-X-NcoI-PNT_F | CCAGTTCAGATCCCTGGACTCCATGGGAGGTTCACAG | Forward primer amplyfing coding sequence of PNT^WT^ or any amb mutant | Linker upstream of PNT |
| D04 | Strep2-X-Bird2_R | CCAGAGGACTTGCCCTCCTTTTCAAACTGGGGGTGGCTC | Reverse primer amplyfing coding sequence of PNT^WT^ or any amb mutant | Strep Linker downstream of PNT |

**Supplementary Table S2: Primer combinations for mRFP1-PNT-sfGFP amber mutant generation for Gibson assembly and PCR product sizes.**

| Name | Primer to produce DNA fragments | DNA Fragment size [bp] |
| --- | --- | --- |
| PNT-WT | C01 + C04 C03 + C02 | 1216 1951 |
| PNT-Q5amb | C01 + C08 C07 + C04 C03 + C02 | 394 865 1951 |
| PNT-S15amb | C01 + C10 C09 + C04 C03 + C02 | 414 845 1951 |
| PNT-Y54amb | C01 + C12 C11 + C04 C03 + C02 | 531 718 1951 |
| PNT-N58amb | C01 + C14 C13 + C04 C03 + C02 | 543 706 1951 |
| PNT-V102amb | C01 + C16 C15 + C04 C03 + C02 | 675 574 1951 |
| PNT-G138amb | C01 + C18 C17 + C04 C03 + C02 | 783 466 1951 |
| PNT-G251amb | C01 + C06 C05 + C04 C03 + C02 | 1121 139 1951 |
| PNT-Q5-S15amb | C01 + C08 C09 + C04 C03 + C02 | 394 845 1951 |
| PNT-N58-V102amb | C01 + C14 C13 + C16 C15 + C04 C03 + C02 | 543 165 574 1951 |
| PNT-Q5-S15-G138amb | C01 + C08 C09 + C18 C17 + C04 C03 + C02 | 394 412 466 1951 |
